# FFT-Based Probability Density Imaging of Euler Solutions

**DOI:** 10.3390/e26060517

**Published:** 2024-06-15

**Authors:** Shujin Cao, Peng Chen, Guangyin Lu, Zhiyuan Ma, Bo Yang, Xinyue Chen

**Affiliations:** 1 School of Earth Sciences and Spatial Information Engineering, Hunan University of Science and Technology, Xiangtan 411201, China; shujin.cao@hnust.edu.cn (S.C.); pengchen@mail.hnust.edu.cn (P.C.); zhiyuanma@mail.hnust.edu.cn (Z.M.); yangbo@mail.hnust.edu.cn (B.Y.); chenxinyue@hnust.edu.cn (X.C.); 2 School of Geosciences and Info-Physics, Central South University, Changsha 410083, China; 3 Institute of Geophysics & Geomatics, China University of Geosciences, Wuhan 430074, China

**Keywords:** Bishop model, Euler deconvolution, B-spline density estimation, probability density imaging, fast linear binning approximation, fast Fourier transform

## Abstract

When using traditional Euler deconvolution optimization strategies, it is difficult to distinguish between anomalies and their corresponding Euler tails (those solutions are often distributed outside the anomaly source, forming “tail”-shaped spurious solutions, i.e., misplaced Euler solutions, which must be removed or marked) with only the structural index. The nonparametric estimation method based on the normalized B-spline probability density (BSS) is used to separate the Euler solution clusters and mark different anomaly sources according to the similarity and density characteristics of the Euler solutions. For display purposes, the BSS needs to map the samples onto the estimation grid at the points where density will be estimated in order to obtain the probability density distribution. However, if the size of the samples or the estimation grid is too large, this process can lead to high levels of memory consumption and excessive computation times. To address this issue, a fast linear binning approximation algorithm is introduced in the BSS to speed up the computation process and save time. Subsequently, the sample data are quickly projected onto the estimation grid to facilitate the discrete convolution between the grid and the density function using a fast Fourier transform. A method involving multivariate B-spline probability density estimation based on the FFT (BSSFFT), in conjunction with fast linear binning appropriation, is proposed in this paper. The results of two random normal distributions show the correctness of the BSS and BSSFFT algorithms, which is verified via a comparison with the true probability density function (pdf) and Gaussian kernel smoothing estimation algorithms. Then, the Euler solutions of the two synthetic models are analyzed using the BSS and BSSFFT algorithms. The results are consistent with their theoretical values, which verify their correctness regarding Euler solutions. Finally, the BSSFFT is applied to Bishop 5X data, and the numerical results show that the comprehensive analysis of the 3D probability density distributions using the BSSFFT algorithm, derived from the Euler solution subset of $\left\{x_{0},y_{0},z_{0}\right\}$, can effectively separate and locate adjacent anomaly sources, demonstrating strong adaptability.

## 1. Introduction

Euler deconvolution is suitable for the semi-automatic estimation of source locations in large-scale potential field data [1,2,3,4,5]. It originates from the overdetermined system of Euler with a large condition number, which causes significant perturbations and often shows divergent trends in Euler solutions [6,7,8,9,10]. Therefore, many researchers use the standard deviation [6] or the truncation error [11] of the overdetermined system of Euler as criteria to filter out spurious solutions. As indicated by FitzGerald et al. [7], correct Euler solutions exhibit the clustering characteristic: “Euler deconvolution yields many similar or duplicate solutions, which may be tightly clustered in the vicinity of real sources.” This is frequently exploited by clustering algorithms to identify anomalous sources. Some additional constraint equations or conditions, such as the horizontal components of the gravity gradient tensor or the relationship between “worming” and Euler equations [12,13], have been proposed for the construction of mixed Euler deconvolution methods [14], seeking to obtain optimal solutions by determining the cluster relationships formed by the Euler solutions and eliminating any spurious solutions that do not belong to a cluster [6,7,10,15,16]. An adaptive fuzzy clustering algorithm has been introduced to address challenges such as the need for predetermined cluster numbers, local optimization in fuzzy clustering analysis, and classification uncertainty [17,18]. However, traditional cluster analysis methods struggle to find the best Euler solution cluster [6,10,14,15].

As a simple application of the clustering characteristic of Euler solutions, the histogram method divides the entire range of structural index values into a series of adjacent and non-overlapping equally spaced bins. It then calculates the number of values in each interval to determine the optimal structural index. This is practical for simple geological structures. However, for complex geological structures, or when there are multiple anomaly sources, it is unrealistic to simply use the histogram method to determine one or more of the “best” structural indices. The density histogram is employed to visualize each formation and to check the coherency of the inversion process [19]. However, it is difficult to efficiently divide a multi-dimensional space into non-coherent histogram bins while keeping the error rates low [20].

A probability density distribution is a continuous version of a histogram with densities, and it is widely used in geophysical inversions. FitzGerald et al. [7] pointed out that the Euler deconvolution yields many similar or duplicate solutions, which may be tightly clustered in the vicinity of real sources, i.e., the 14th strategy in the paper [7]. Based on this strategy, Cao et al. [21] proposed a non-parametric estimation method based on the normalized B-spline probability density (BSS), aiming to separate the Euler solution clusters to mark different anomaly sources according to the similarity and density characteristics of the Euler solutions [22,23]. B-spline curves are linear combinations of B-spline basis functions connected by node vectors [24]. The *c* + 1 order B-spline basis functions can all be obtained recursively from the lower-order basis functions [25]. Faenza et al. [26] developed a novel non-parametric multivariate model to depict the spatial and temporal distributions of large earthquakes. The model enables the intuitive examination of diverse hypotheses, including all forms of time dependency (e.g., seismic gaps, clustering, and Poisson assumptions) [27,28]. Mautz et al. [29] proposed the so-called B-spline wavelets to represent the spatial time series of the GPS-derived global ionosphere maps of the vertical total electron content of the Earth’s surface to the mean altitudes of GPS satellites over Japan. Herrmann and Hennenfent [30] proposed a recovery method based on non-parametric transformation, utilizing recently developed curvelet frames to compress seismic data [31].

To display and calculate the probability density value of the sample, it is generally necessary to construct an estimation grid, which consists of a set of equidistant or non-equidistant spatial points and generally needs to cover the entire input sample. However, when the data sample is large or the estimation grid is extensive, the BSS algorithm, using brute force computation [32,33], i.e., traversing each grid node over the data sample, becomes computationally intensive and consumes excessive amounts of memory. Wang et al. [34] proposed a flexible and high-precision algorithm to perform transformations of the potential field and gradient components in 3D and 2D, as well as derivative calculations in the spatial domain using cubic B-splines, applicable at any point within the computational domain. To reduce the computational intensity of kernel density estimation, Wand [32] proposed the binned kernel density estimation, which first uses the fast linear binning technique to map the sample data onto the predefined estimation grid and then obtains the grid counts of the sample data concerning the nodes of the estimation grid, to compute the approximate value of the kernel estimation on the estimation grid over the sample data using the fast Fourier transform (FFT) [35,36]. Based on Wand [32], Cao et al. [37] focused on the computational details of the sample projection in the estimation grid. Based on fast linear binning approximation and Fourier-based fast convolution, the multivariate kernel density derivative estimation (KDDE) was proposed to compute the probability values of Euler solutions derived from tensor gravity data using tensor Euler deconvolution. The algorithm is an extension of kernel density estimation [38], which is a mathematical process used to estimate the probability density function of a random variable. The principle and procedure of the fast linear binning approximation, Fourier-based convolution, and the KDDE algorithm for computing the probability values of Euler solutions were detailed. Then, the effects of the bandwidths and the estimation grid’s size on the computational efficiency of fast linear binning approximation and the KDDE algorithm were analyzed. The results obtained from synthetic models and field data showed that the multivariate KDDE, in conjunction with fast linear binning approximation and the FFT, demonstrated high computational efficiency to overcome this difficulty. Moreover, the resulting probability density isosurface successfully locates meaningful geological targets.

This paper proposes a B-spline probability density estimation method based on the FFT (BSSFFT) in conjunction with a fast linear binning approximation, aiming to rapidly map Euler solutions to the estimation grid. Using a fast Fourier transform, the discrete convolution of the estimation grid and the density function is achieved. Compared with the BSS, BSSFFT can effectively avoid the issue of brute force calculation. Simultaneously, it provides a more focused probability density estimation result for identifying various anomaly sources. Numerical tests and synthetic models demonstrate the reliability of the BSS and BSSFFT. Finally, the algorithm was applied to magnetic field data from the Bishop model, which was generated from topographic data from a portion of the volcanic tablelands area north of Bishop, California. The probability density isosurfaces of the synthetic models and the field data obtained by BSSFFT distinguish the clusters formed by the Euler solutions, effectively separating and locating adjacent anomaly sources.

## 2. Theory

### 2.1. Principle of Gravity Euler Deconvolution

Following the suggestions of Reid [39] and based on the 14th strategy proposed by FitzGerald et al. [7], the Euler deconvolution can be expressed as follows:

(1) $$x_{0}\frac{\partial g_{z}}{\partial x}+y_{0}\frac{\partial g_{z}}{\partial y}+z_{0}\frac{\partial g_{z}}{\partial z}+N\left(B_{z}-g_{z}\right)=x\frac{\partial g_{z}}{\partial x}+y\frac{\partial g_{z}}{\partial y}+z\frac{\partial g_{z}}{\partial z}$$

where $\alpha \in \left\{x,y,z\right\}$; $B_{z}$ is the anomalous background field; $g_{z}$ is the gravity field; and ∂gz∂gz∂α∂β is the partial derivative of $g_{z}$ with respect to β. (x,y,z) are the coordinates of the observation point; and $\left(x_{0},y_{0},z_{0}\right)$ are the spatial positions of the sought anomaly source. When the term regarding $\frac{\partial g_{z}}{\partial y}$ is removed, Equation (1) can be applied to the 2D profile data used to obtain $\left\{x_{0},z_{0},N\right\}$. The structural index *N* depends on the nature (type) of the geologic source, which makes it challenging to characterize multiple anomalous sources using a single structural index [40,41]. Due to the difficulty in determining the *N* of the geological target of interest [7,42], the traditional Euler deconvolution uses a series of predefined structural indices and yields many spurious solutions [10]. Many researchers have attempted to determine the best cluster via manual evaluation using a series of tentative *N* values, which significantly increases the interpreter’s workload, particularly for complex geological structures with multiple anomalous sources [6,10,43,44,45]. To overcome these problems, *N* will not be predicted in this paper [6,7,42].

Utilizing a square sliding window of size $w_{x}$=$w_{y}$=*w* to gradually traverse the entire survey area, and using Equation (1), each sliding window can form an equation system, as follows:

(2) $$\left[\begin{array}{cccc} {\left(\partial g_{z}/\partial x\right)}^{1} & {\left(\partial g_{z}/\partial y\right)}^{1} & {\left(\partial g_{z}/\partial z\right)}^{1} & {\left(g_{z}-B_{z}\right)}^{1}\\ \cdots & \cdots & \cdots & \cdots \\ {\left(\partial g_{z}/\partial x\right)}^{j} & {\left(\partial g_{z}/\partial y\right)}^{j} & {\left(\partial g_{z}/\partial z\right)}^{j} & {\left(g_{z}-B_{z}\right)}^{j}\\ \cdots & \cdots & \cdots & \cdots \\ {\left(\partial g_{z}/\partial x\right)}^{n_{w}} & {\left(\partial g_{z}/\partial y\right)}^{n_{w}} & {\left(\partial g_{z}/\partial z\right)}^{n_{w}} & {\left(g_{z}-B_{z}\right)}^{n_{w}} \end{array}\right]\left[\begin{array}{c} x_{0}^{i}\\ y_{0}^{i}\\ z_{0}^{i}\\ N^{i} \end{array}\right]=\left[\begin{array}{c} b_{z}^{1}\\ \cdots \\ b_{z}^{j}\\ \cdots \\ b_{z}^{n_{w}} \end{array}\right]$$

where $b_{z}^{j}={\left(x\partial g_{z}/\partial x\right)}^{j}+{\left(y\partial g_{z}/\partial y\right)}^{j}+{\left(z\partial g_{z}/\partial z\right)}^{j}$, $n_{w}=w_{x}\times w_{y}$; the Euler deconvolution solution set is denoted as $m=\{m^{i}\}$; and the *i*-th Euler deconvolution solution is denoted as $m^{i}={\left[x_{0}^{i},y_{0}^{i},z_{0}^{i},N_{0}^{i}\right]}^{T}$, representing the spatial location and structural indices of the sought anomaly source. The superscript T denotes the transpose operator.

### 2.2. Principle of BSS for Euler Solutions

Traditional Euler deconvolution optimization strategies are not able to differentiate optimal solutions from pseudo-traces (misplaced solutions, which must be removed or marked) among Euler solutions. Therefore, the BSS algorithm, which is based on normalized B-splines, is adopted to separate each cluster based on the similarity and clustering degree of the Euler solutions in order to identify various sources of anomalies [7]. To introduce the BSS algorithm [21], let $X^{1},\cdots ,X^{i},\cdots ,X^{n}$ be an independent and identically distributed random sample of a *d*-dimensional probability density function (pdf) *f*. The sample *i*th can be represented as $X^{i}={\left[X_{1}^{i},\cdots ,X_{j}^{i},\cdots ,X_{d}^{i}\right]}^{T}$. Specifically, $X^{i}$ can denote different combinations of Euler solutions, $x_{0}^{i}$, $y_{0}^{i}$, $z_{0}^{i}$, $N^{i}$, such as $x_{0}^{i}$, ${\left[x_{0}^{i},y_{0}^{i}\right]}^{T}$, ${\left[x_{0}^{i},y_{0}^{i},z_{0}^{i}\right]}^{T}$, ${\left[x_{0}^{i},y_{0}^{i},z_{0}^{i},N^{i}\right]}^{T}$, 1≤d≤4.

Projecting samples $X^{i}$ onto an estimation grid $\chi ={\left[\chi_{1},\cdots ,\chi_{j},\cdots ,\chi_{d}\right]}^{T}$ involves a grid designed as a unified structure to accurately cover the input samples, with a size of $\lambda ={\left[\lambda_{1},\cdots ,\lambda_{j},\cdots ,\lambda_{d}\right]}^{T}$, a bandwidth of $h={\left[h_{1},\cdots ,h_{j},\cdots ,h_{d}\right]}^{T}$, and upper and lower bounds of $\left\{a_{j}\right\}$/$\left\{b_{j}\right\}$. The relationship between these variables is as follows:

(3) $$h_{j}=\left(b_{j}-a_{j}\right)/\left(\lambda_{j}-1\right)$$

On an estimate point $\bar{x}=\left[{\bar{x}}_{1},\cdots ,{\bar{x}}_{j},\cdots ,{\bar{x}}_{d}\right]$ of the estimation grid χ, for a sample $X^{i}$, the estimation of the probability density of the *d*-dimensional B-spline can be expressed using *c* uniform B-spline functions $B_{l_{j}}^{c}\left(\bar{x}\right)$, denoted as $\hat{f}$, given by the following Gehringer and Redner [46]:

(4) $$\hat{f}\left(\bar{x}\right)=\sum_{l_{1}=1}^{\lambda_{1}}\cdots \sum_{l_{d}=1}^{\lambda_{d}}\frac{a_{l_{1}\cdots l_{j}\cdots l_{d}}}{h_{1}\cdots h_{j}\cdots h_{d}}B_{l_{1}}^{c}\left({\bar{x}}_{1}\right)\cdots B_{l_{j}}^{c}\left({\bar{x}}_{j}\right)\cdots B_{l_{d}}^{c}\left({\bar{x}}_{d}\right)$$

Where the subscript $l_{j}$ specifies a position along the *j*th dimension of the estimation grid χ, and the coefficient $a_{l}=a_{l_{1}\cdots l_{j}\cdots l_{d}}$ in the equation can be expressed by sample $X^{i}$ as follows:

(5) $$a_{l_{1}\cdots l_{j}\cdots l_{d}}=\frac{1}{n}\sum_{i=1}^{n}B_{l_{1}}^{c}\left(X_{1}^{i}\right)\cdots B_{l_{j}}^{c}\left(X_{j}^{i}\right)\cdots B_{l_{d}}^{c}\left(X_{d}^{i}\right)$$

where, for sample $X^{i}$, based on the normalized *c*th-order uniform B-spline $B^{c}$ and the *j*th-dimensional bandwidth $h_{j}$, the B-spline basis functions $B_{l_{j}}^{c}\left({\bar{x}}_{j}\right)$ can be redefined as follows:

(6) $$B_{l_{j}}^{c}\left({\bar{x}}_{j}\right)\equiv B_{l_{j}}^{c}\left(X_{j}^{i}\right)=B^{c}\left(\frac{X_{j}^{i}}{h_{j}}-\left\lfloor \frac{X_{j}^{i}}{h_{j}}\right\rfloor \right)$$

where $B^{c}$ is a normalized $c^{th}$-order uniform B-spline.

**Theorem** **1.** *The uniform B-splines, $\left\{B_{k}^{c}\left(\bar{x}\right)\right\}$, form a partition of unity* [47]*, i.e.,*

(7) $$\sum_{k=-\infty }^{\infty }B_{k}^{c}\left(\bar{x}\right)=\sum_{k=j-c+1}^{j}B_{k}^{c}\left(\bar{x}\right)=1$$

*for all $\bar{x}\in \left[{\bar{x}}_{j},{\bar{x}}_{j+1}\right]$.*

where c=1, 2, and 3, $B^{c}\left(\bar{x}\right)$ in Equation (6) can be expressed as follows:

(8) $$B^{1}\left(\bar{x}\right)=\left\{\begin{array}{cc} \bar{x}, & \;0\leq \bar{x}<1\\ (2-\bar{x}), & \;1\leq \bar{x}\leq 2 \end{array}\right.$$

(9) $$B^{2}\left(\bar{x}\right)=\left\{\begin{array}{cc} {\bar{x}}^{2}/2, & \;0\leq \bar{x}<1\\ \left(-2{\bar{x}}^{2}+6\bar{x}-3\right)/2, & \;1\leq \bar{x}<2\\ {\left(3-\bar{x}\right)}^{2}/2, & \;2\leq \bar{x}\leq 3 \end{array}\right.$$

(10) $$B^{3}\left(\bar{x}\right)=\left\{\begin{array}{cc} {\bar{x}}^{3}/6, & \;0\leq \bar{x}<1\\ \left(-3{\bar{x}}^{3}+12{\bar{x}}^{2}-12\bar{x}+4\right)/6, & \;1\leq \bar{x}<2\\ \left(3{\bar{x}}^{3}-24{\bar{x}}^{2}+60\bar{x}-44\right)/6, & \;2\leq \bar{x}<3\\ \left(-{\bar{x}}^{3}+12{\bar{x}}^{2}-48\bar{x}+64\right)/6, & \;3\leq \bar{x}\leq 4 \end{array}\right.$$

### 2.3. Principle of BSSFFT

Through Equation (4), the probability density values of each Euler solution can be calculated on the estimation grid χ. To overcome the issue of brute force computations [37,48], an improved BSS method is proposed based on fast linear binned approximation [32]. Equation (4) can be reconstructed as follows:

(11) $$\hat{f}\left(\chi_{j}\right)=n^{-1}\sum_{l_{1}=1}^{\lambda_{1}}\cdots \sum_{l_{d}=1}^{\lambda_{d}}B_{K}^{c}\left(\chi_{j}^{l_{j}}-\chi_{l}^{l_{j}}\right)C_{l}$$

where $C_{l}$ is the grid count obtained via approximate fast binning at point $\chi_{l}$ of the estimation grid, $1\leq l_{j}\leq \lambda_{j}$.

To compute $C_{l}$ in Equation (11), we define it in the *j*-dimensional direction with edge $\left[\chi_{j}^{l_{j}},\chi_{j}^{l_{j+1}}\right]$ and denote it as $\chi_{j}^{l_{j},l_{j}+1}$. Assume that $X^{i}$ is precisely located in a hyper-rectangle (also called a bin) *V* formed by the edges $\chi_{1}^{l_{1},l_{1}+1},\cdots ,\chi_{j}^{l_{j},l_{j}+1},\cdots \chi_{d}^{l_{d},l_{d}+1}$, with $2^{d}$ nodes and $2^{d}$ faces [49]. Then, the projection of $X_{j}^{i}$ on $\chi_{j}^{l_{j},l_{j}+1}$ can be used to represent weights $u_{j}^{0}$ and $u_{j}^{1}=1-u_{j}^{0}$ at both ends of edge $\chi_{j}^{l_{j},l_{j}+1}$, as follows:

(12) $$u_{j}^{0}=\frac{X_{j}^{i}}{h_{j}}-\left\lfloor \frac{X_{j}^{i}}{h_{j}}\right\rfloor \equiv \frac{X_{j}^{i}-\chi_{j}^{l_{j}}}{h_{j}}$$

where ⌊⌋ represents the floor operation.

In other words, $u_{j}^{0}$ and $u_{j}^{1}$ are equivalent to the natural coordinate values of $X^{i}$ induced by the two endpoints of edge $\chi_{j}^{l_{j},l_{j}+1}$. Taking a vertex *v* of *V* as an example, according to Rao [50] and Chacón and Duong [49], the corresponding grid count ${\bar{C}}_{l}$ of sample $X^{i}$ is equivalent to the multiplication of the natural coordinate values ($u_{j}\in \left\{u_{j}^{0},u_{j}^{1}\right\}$) induced by $X^{i}$ with respect to the adjacent edges of the vertex of *V*, which means that ${\bar{C}}_{l}$ is equal to the natural coordinate values of *v* with respect to $X^{i}$. ${\bar{C}}_{l}$ can be expressed as follows:

(13) $${\bar{C}}_{l}=\prod_{j=1}^{d}u_{j}$$

**Figure 1:**
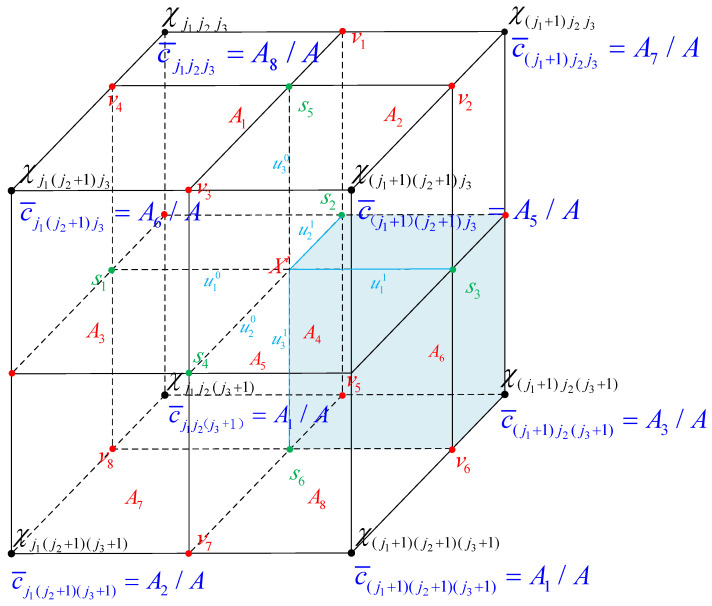
Linear binning counts: a trivariate datum $\chi^{i}$ is converted into the counts assigned to its eight nearest grid points. Following Rao’s work [50] and Chacón and Duong’s work [49], their respective counts are equal to their natural coordinate values.

As shown in Figure 1, suppose that a trivariate point $X^{i}$ is located exactly in a bin surrounded by 8 points $\chi_{j_{1}j_{2}j_{3}}$, ⋯,$\chi_{\left(j_{1}+1\right)\left(j_{2}+1\right)\left(j_{3}+1\right)}$. To obtain the count ${\bar{C}}_{l}$ of the point with respect to its 8 neighboring vertices, we project sample $X^{i}$ onto the 8 edges and 6 faces of the bin, and the points on the edges $v_{1},v_{2},\cdots ,v_{8}$ and the points on the faces $s_{1},s_{2},\cdots ,s_{6}$ are obtained. Then, three lines, $s_{1}s_{3},s_{2}s_{4}$, and $s_{5}s_{6}$, are formed to pass through sample $X^{i}$. Using planes parallel to the coordinate axes and passing through these lines, the bin is divided into sub-bins $A_{1},\cdots ,A_{8}$ from top to bottom in a clockwise direction. Taking the point $\chi_{j_{1}\left(j_{2}+1\right)j_{3}}$ as an example, according to Equation (12), the lines $s_{1}s_{3},s_{2}s_{4}$, and $s_{5}s_{6}$ are each divided into two segments and labeled $u_{1}^{0},u_{1}^{1},u_{2}^{0},u_{2}^{1},u_{3}^{0}$, and $u_{3}^{1}$, respectively. Thus, according to Equation (13), the grid count of $\chi_{j_{1}\left(j_{2}+1\right)j_{3}}$ with respect to $X^{i}$ is as follows:

(14) $${\bar{C}}_{l}=\prod_{j=1}^{d}u_{j}=u_{1}^{1}u_{2}^{1}u_{3}^{1}=A_{6}$$

Thus, when a sample $X^{i}$ falls within a bin, for a node *v* of the bin, its corresponding grid count with respect to the sample is determined by the length (in 1D), area (in 2D), or volume (in 3D) that the diagonal node from this node *v* encloses with the sample, as shown in Figure 1. For the calculation of the count ${\bar{C}}_{l}$ at an estimated point for univariate and bivariate $X^{i}$, please see Cao et al. [37]. For each estimation point $\chi_{l}$, its grid count $C_{l}$ is the sum of the grid counts ${\bar{C}}_{l}$ of all samples pertaining to it.

According to the algorithm of Wand [32], Equation (11) can be rewritten in the form of convolution, as follows:

(15) $${\hat{f}}_{l}=\sum_{k_{1}=-\lambda_{1}+1}^{\lambda_{1}-1}\cdots \sum_{k_{j}=-\lambda_{j}+1}^{\lambda_{j}-1}\cdots \sum_{k_{d}=-\lambda_{d}+1}^{\lambda_{d}-1}C_{l-k}r_{k}$$

where

(16) $$r_{k}=\frac{1}{n}B_{k}^{c}\left(h_{1}k_{1},\cdots ,h_{j}k_{j},\cdots ,h_{d}k_{d}\right)$$

where $k_{j}$ is the value of *f* along the *j* dimension of the estimation grid, and the convolution of *C* and *r* in Equation (15) can be computed using the discrete convolution theorem. When matrices *C* and *r* differ in size, techniques such as zero-padding and wrap-around ordering (see Chapter 13 of Teukolsky et al. [51]) are used to ensure that *C* and *r* match in size, leveraging the special structured matrix properties discussed in [51,52]. For ease of understanding, only the 2D scenario, as expressed in Equations (17) and (18), is provided here.

(17) $$C=\left[\begin{array}{ccccc} C_{1,1} & C_{1,2} & \cdots & C_{1,\lambda_{2}} & \\ \vdots & \vdots & \ddots & \vdots & 0\\ C_{\lambda_{1},1} & C_{\lambda_{1},2} & \cdots & C_{\lambda_{1},\lambda_{2}} & \cdots \\ \; & 0 & \; & \vdots & 0 \end{array}\right]$$

(18) $$r=\left[\begin{array}{ccccccccc} r_{0,0} & r_{0,1} & \cdots & r_{0,\lambda_{2}} & \; & r_{0,\lambda_{2}} & \cdots & r_{0,1}\\ r_{1,0} & r_{1,1} & \cdots & r_{1,\lambda_{2}} & \; & r_{1,\lambda_{2}} & \cdots & r_{1,1}\\ \vdots & \vdots & \ddots & \vdots & 0 & \vdots & \ddots & \vdots \\ r_{\lambda_{1},0} & r_{\lambda_{1},1} & \cdots & r_{\lambda_{1},\lambda_{2}} & \cdots & r_{\lambda_{1},\lambda_{2}} & \cdots & r_{\lambda_{1},1}\\ & 0 & \; & \vdots & 0 & \vdots & 0 & \; & \\ r_{\lambda_{1},0} & r_{\lambda_{1},1} & \cdots & r_{\lambda_{1},\lambda_{2}} & \cdots & r_{\lambda_{1},\lambda_{2}} & \cdots & r_{\lambda_{1},1}\\ \vdots & \vdots & \ddots & \vdots & 0 & \vdots & \ddots & \vdots \\ r_{1,0} & r_{1,1} & \cdots & r_{1,\lambda_{2}} & \; & r_{1,\lambda_{2}} & \cdots & r_{1,1} \end{array}\right]$$

Then, *C* and *r* will have the same dimensions as $\lambda_{1}\times ,\cdots ,\times \lambda_{j}\times ,\cdots ,\times \lambda_{d}$. According to the discrete convolution theorem [32], Equation (19) can be re-expressed as follows:

(19) $$S=F^{-1}\left(F\left(C\right)F\left(r\right)\right)$$

where *F* and $F^{-1}$ are denoted as the Fourier transform and the Fourier inverse transform.

The sought density estimate $\hat{f}$ can be obtained by dividing the submatrix of *S* in Equation (19) by the product of $\lambda_{j},j\in \left\{1,\cdots ,d\right\}$.

(20) $$\hat{f}=\prod_{j=1}^{d}\lambda_{j}^{-1}S\left[1:\lambda_{1},\cdots ,1:\lambda_{d}\right]$$

where for the 2D case, *S*[a:b,c:d] represents the subset of rows from *a* to *b* and the submatrix of columns from *c* to *d* for matrix *S*.

The BSSFFT technique first converts the samples into grid counts at the estimation grid points using fast linear binning approximation via the FFT. Referring to the works of Wand [32], Raykar et al. [33], the computational complexity of brute force computation is $O\left(n^{2}\right)$, and the computational complexity of the algorithm described in this paper is only $O\left(\prod_{i=1}^{d}\lambda_{i}\log\left(\prod_{i=1}^{d}\lambda_{i}\right)\right)$. Cao et al. [37] provided a detailed analysis of the computational performance of fast linear binning approximation via the FFT for the KDDE algorithm, which is in line with the expectations regarding computational complexity described in [33]. Moreover, interested readers can refer to the relevant open-source codes, such as KS [48], KDEpy [53], and Vikas [54], to build their own applications. All the tests in this paper were carried out on a server equipped with an Intel(R) Xeon(R) Gold 5117 CPU and 256 GB of memory.

## 3. Algorithm Verification

A 1D random dataset with a sample size of n=3000 was constructed using three normal distributions with a mean of $3^{-1}\varphi \left(0,0.25\right)+3^{-1}\varphi \left(5,0.5\right)+3^{-1}\varphi \left(11,0.75\right)$. The Gaussian kernel smoothing estimation algorithm (KS) [55,56] is introduced as a comparison to validate the reliability of the BSS and BSSFFT algorithms.

**Figure 2:**
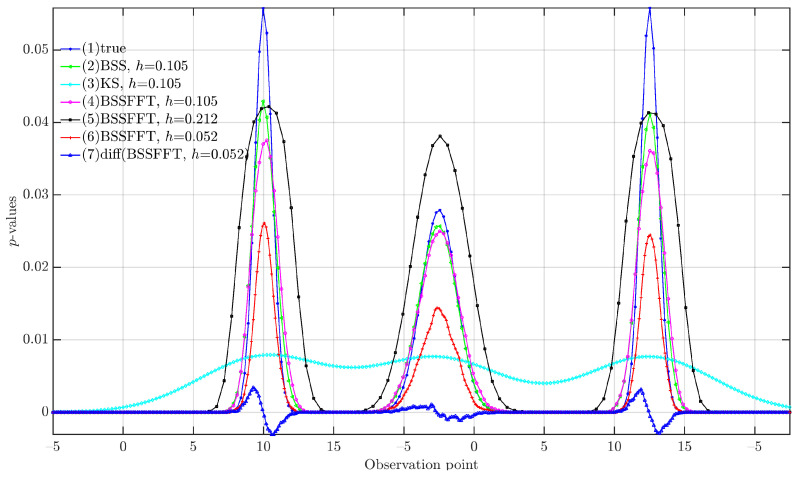
The 1D verification results. BSS: B-spline probability density estimation method; KS: Gaussian kernel smoothing estimation method; BSSFFT: B-spline probability density estimation method based on fast Fourier transform.

In Figure 2, the BSS (Curve 2), KS (Curve 3), and BSSFFT (Curves 4–6) methods all exhibit three probability peaks at the same positions, with peak coordinates of 0, 5, and 11, consistent with the theoretical parameters of three normal distributions. Under the same bandwidth conditions, compared to the true pdf (Curve 1), the KS estimate is too smooth and fails to provide indicative information; the BSS estimate approximates the true pdf, demonstrating the correctness of the BSS algorithm; and the BSSFFT results differ slightly from the true pdf, but the difference is insignificant. It arises from the approximation process of obtaining the grid counts on the estimation grid, which utilizes the fast linear binning approximation method with the samples. In Curves 4–6, the results were obtained using the default bandwidth (Curve 4), which closely approximates the true probability density function (pdf). Using a large bandwidth results in an estimate that is larger than the true pdf, due to the small number of estimation grid points. This result is affected by probability density normalization, which inflates the probability values at the estimation points. Conversely, with a small bandwidth—that is, a large number of estimation grid points under the same input sampling conditions—the probability density normalization causes the values at the estimation points to be deflated. By examining the differences in the estimation results with a small bandwidth (Curve 6), it is evident that the zero points in Curve 7, which are derived from the differences in Curve 6, align precisely with the theoretical positions of normal distributions in the artificially generated sample data. This demonstrates that the proposed BSSFFT algorithm effectively reflects the distribution of the true pdf.

Furthermore, a random 2D dataset with a sample size of *n*= 30,000 is generated using three normal distributions with 10,000 data points. The specific random normal distribution functions are as follows:

(21) $$f\left(x\right)=\frac{1}{3}\sum_{i=1}^{3}\varphi \left(\mu_{i},\sigma_{i}\right)$$

The parameters of each normal distribution of Equation (21) are as follows:

(22) $$\begin{array}{c} \mu_{1}=\left(\begin{array}{c} 0\\ 0 \end{array}\right),\mu_{2}=\left(\begin{array}{c} 5\\ -5 \end{array}\right),\mu_{3}=\left(\begin{array}{c} -5\\ 5 \end{array}\right)\\ \sigma_{1}=\left(\begin{array}{cc} 0.75 & -0.5\\ -0.5 & 0.75 \end{array}\right),\sigma_{i}=\left(\begin{array}{cc} 0.75 & 0.5\\ 0.5 & 0.75 \end{array}\right),i\in \left\{2,3\right\} \end{array}$$

**Figure 3:**
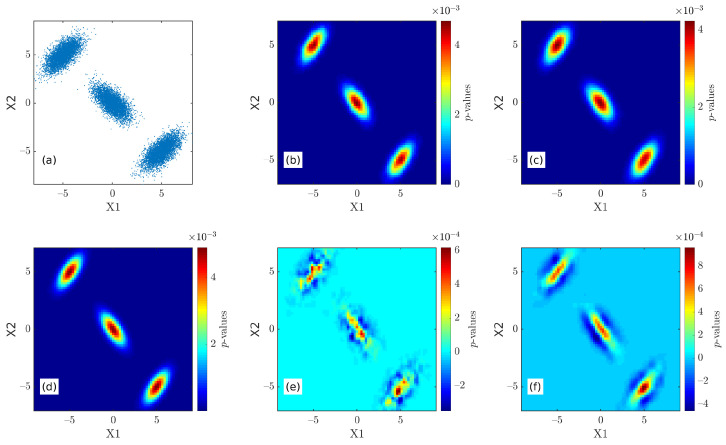
The 2D verification results: (**a**) the 2D random dataset, n=3000; (**b**) the BSS result; (**c**) the BSSFFT result; (**d**) the true probability density function (pdf); (**e**) the relative error between the BSS result and true pdf; (**f**) the relative error between the BSSFFT result and true pdf.

As shown in Figure 3, the 2D BSS results all show three probability density peaks whose horizontal coordinates are (0,0), (5,−5), and (−5,5), which are consistent with the settings of Equation (22). The results of both the BSS and BSSFFT methods are not significantly different from the theoretical values; however, the results of BSSFFT are smoother than those of the BSS method due to the use of fast linear binning approximation. The reliability of the BSS and BSSFFT algorithms can be verified by analyzing the results of the random 1D and 2D normal distribution data.

## 4. Model Experiment

### 4.1. Single Cubic Model

A homogeneous cube has analytical solutions and its theoretical structural index is 2 [21,57]. Therefore, a single cube model with a center (−1000,−2000,1500), a size of 2000 m × 2000 m × 2000 m, and a residual density of 0.36 g/cm^3^ is constructed to further test the adaptability of the B-spline density estimation to Euler solutions and the reliability of the algorithm. The observation height is 50 m; the range of the survey area is *x*: 0 m∼10,000 m, *y*: 0 m∼10,000 m; and the size of the survey grid is 100 m × 100 m. The forward data are contaminated by Gaussian random noise with a zero mean and a standard deviation of $\sigma (\bar{p})$:

(23) $$\sigma \left(\bar{p}\right)=\left(\max\left(d_{obs}\right)-\min\left(d_{obs}\right)\right)\times \bar{p}$$

where the min and max functions return the minimum and maximum values of the input data.

**Figure 4:**
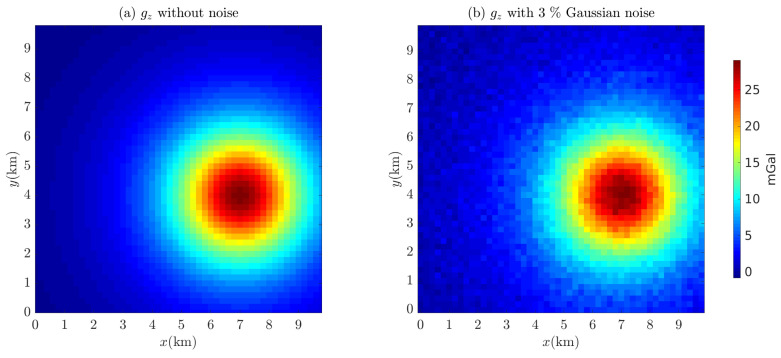
$g_{z}$ of the cubic model (**a**) without noise and (**b**) with 3% Gaussian noise. The residual density of the cube is 0.36 g/cm^3^.

**Figure 5:**
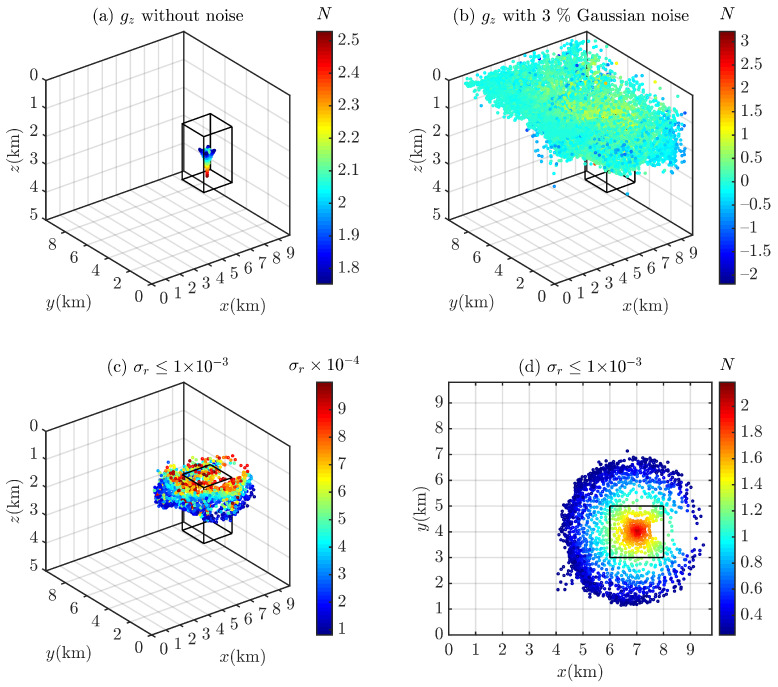
Scatter plots of Euler solutions. (**a**) $g_{z}$ without noise and (**b**) with 3% Gaussian noise, Euler solutions filtered by $\sigma_{r}\leq 1\times 10^{-3}$ [4] in different views: (**c**) (−37.5,30) and (**d**) (90,0).

Euler deconvolution with a sliding window of 15 × 15 grid points is used to process the contaminated gravity data ($\bar{p}=3\%$; see Figure 4) to obtain the Euler solutions, which contain structural indices and spatial positions $\left(x_{0},y_{0},z_{0}\right)$. In this paper, all Euler solutions that are outside the moving window width or the observed measurement data, N<0 or N>3, are eliminated. A total of 4112 Euler solutions remain after filtering. Figure 5 shows the scatter plots of the Euler solutions. The Euler deconvolution yields very pure solutions when there is no noise in the data (see Figure 5a); in contrast, as the Euler equations are easily affected by noise, the Euler solutions tend to diverge when there is noise in the data (see Figure 5b). For this reason, we use an empirical formula proposed by Thompson [4] to cull out spurious solutions [4,6]:

(24) $$\sigma_{r}=N\frac{\sigma }{z_{0}}$$

where $z_{0}$ is the estimated depth and $\sigma_{r}$ is the standard deviation $z_{0}$ of the solution σ (i.e., *m*) relative to the estimated depth. Most of the spurious solutions are eliminated when $\sigma \leq 10^{-3}$, as shown in Figure 5c,d.

**Figure 6:**
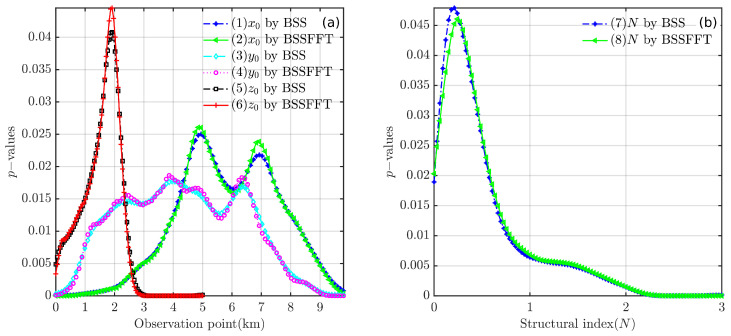
Probability density curves obtained using 1D BSS and BSSFFT with λ=100 for subsets of the Euler solutions derived from noise-corrupted data. (**a**) subsets $\left\{x_{0}\right\}$, $\left\{y_{0}\right\}$, and $\left\{z_{0}\right\}$; (**b**) subset {N}.

Regarding the computation time for the analysis of one-dimensional subsets $\left\{x_{0}\right\}$, $\left\{y_{0}\right\}$, $\left\{z_{0}\right\}$, and *N*, the BSS algorithm requires 0.1083, 0.0817, 0.0788, and 0.1011 s, and the BSSFFT algorithm requires 0.05667, 0.0338, 0.0329, and 0.0334 s, respectively. Figure 6 shows the probability curves derived from subsets of Euler solutions using the 1D BSS and BSSFFT methods with the same bandwidth conditions. It is clear from the curves in Figure 6 that both 1D BSS and BSSFFT show multiple peaks in the estimation results for subsets $\left\{x_{0}\right\}$, $\left\{y_{0}\right\}$, $\left\{z_{0}\right\}$, and {N}. When analyzing Figure 5 and Figure 6 simultaneously, we note that the rightmost peaks of curves 1–2 and 5–6 in Figure 6 are correlated with the *x*/*z* coordinates of the anomaly source center, while curves 3–4 show multiple peaks, but it is not possible to determine which peak corresponds to the *y* coordinate of the anomaly source center, and due to the excessive number of spurious solutions, the peaks correlated with the theoretical value of *N* are not efficiently extracted. Although the abscissas of some peaks in the curves of Figure 6 are partially consistent with the model settings, the estimation results of the 1D subset all exhibit multiple peaks. This increases the difficulty in manually selecting peaks and is likely to cause ambiguity. The 4D set $\left\{x_{0},y_{0},z_{0},N\right\}$ of the single cube model is now arranged into six groups of 2D subsets: $\left\{x_{0},y_{0}\right\}$, $\left\{x_{0},z_{0}\right\}$, $\left\{y_{0},z_{0}\right\}$, $\left\{x_{0},N_{0}\right\}$, $\left\{y_{0},N_{0}\right\}$, $\left\{z_{0},N_{0}\right\}$. A total of 12 results are obtained using the 2D BSS and BSSFFT for all Euler solution subsets.

**Figure 7:**
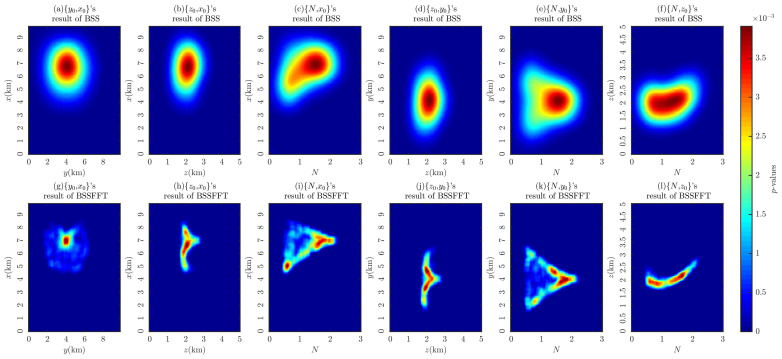
Probability density distribution obtained using 2D BSS and BSSFFT with $\lambda =\left[100,100\right]$ for subsets $\left\{x_{0},y_{0}\right\}$, $\left\{x_{0},z_{0}\right\}$, $\left\{y_{0},z_{0}\right\}$, $\left\{x_{0},N\right\}$, $\left\{y_{0},N\right\}$, and $\left\{z_{0},N\right\}$ of the cubic model.

Figure 7 shows the probability density distributions obtained through the 2D BSS and BSSFFT analysis of 2D subsets for a single cubic model. Regarding the computation time for the analysis of the 2D subsets $\left\{x_{0},y_{0}\right\}$, $\left\{x_{0},z_{0}\right\}$, $\left\{y_{0},z_{0}\right\}$, $\left\{x_{0},N\right\}$, $\left\{y_{0},N\right\}$, and $\left\{z_{0},N\right\}$, the BSS algorithm requires 0.5212, 0.4752, 0.5231, 0.4862, 0.5186, and 0.5102 s, while the BSSFFT algorithm requires 0.0423, 0.0397, 0.0425, 0.0420, 0.0436, and 0.0413 s, respectively. The values of $x_{0}$, $y_{0}$, $z_{0}$, and *N* corresponding to the main probability density peaks of each subplot are 7000 m, 4000 m, 2000 m, and ≈1.8, respectively, due to the presence of a large number of spurious solutions near z=0 and N=0 in the shallow part of Figure 5. This leads to the appearance of peaks with small tails, which correspond to misplaced solutions, in each subplot, with the exception of Figure 7a,g. For example, see Figure 7c–f,h–i. By comparing the probability densities of the subplots in Figure 7a,g,c,i,e,k with those in Figure 7f,i, it can be seen that BSSFFT effectively eliminates spurious solutions and determines the anomalous source centers by converting the samples into grid counts through fast linear binning approximation. Compared to the 1D results, the 2D results of BSS and BSSFFT show multiple peaks for both the depth and structural index estimation of the model, but only one peak is dominant, providing a basis for the determination of the *N* and spatial locations of the anomalies. When analyzing the presence of multiple peaks, the equivalence effect [58] is considered, i.e., two adjacent cubes can also produce such anomalies, but there is some ambiguity [37]. When analyzing subsets, the probability density trailing phenomenon is more obvious. Furthermore, the graphical interpretation of the 5D results of the 4D probability density estimation is too complex. To solve these problems, four 3D subsets, $\left\{x_{0},y_{0},z_{0}\right\}$, $\left\{x_{0},y_{0},N\right\}$, $\left\{x_{0},z_{0},N\right\}$, and $\left\{y_{0},z_{0},N\right\}$, are derived from the entire Euler solution set $\left\{x_{0},y_{0},z_{0},N\right\}$ and analyzed using the 3D BSS and BSSFFT.

**Figure 8:**
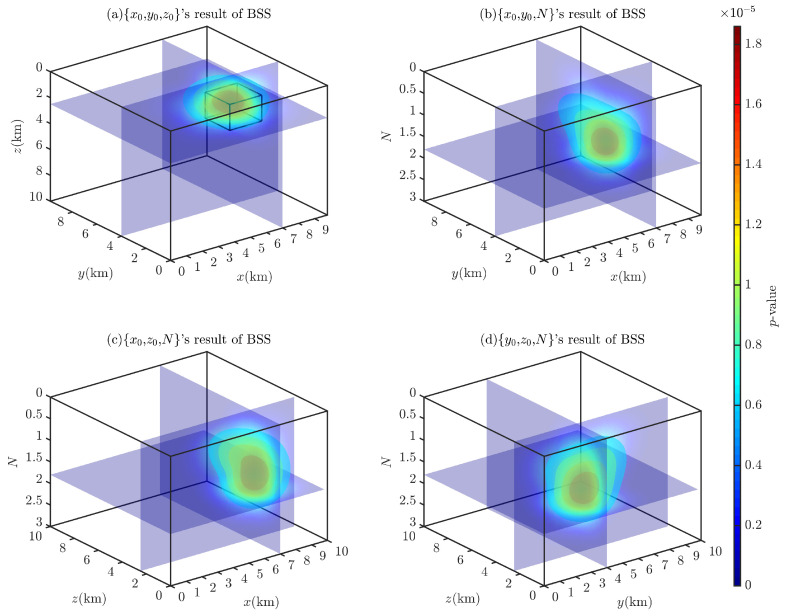
Probability density isosurface obtained using 3D BSS with $\lambda =\left[100,100,100\right]$ for Euler solution subsets: (**a**) $\left\{x_{0},y_{0},z_{0}\right\}$; (**b**) $\left\{x_{0},y_{0},N\right\}$; (**c**) $\left\{x_{0},z_{0},N\right\}$; (**d**) $\left\{y_{0},z_{0},N\right\}$.

**Figure 9:**
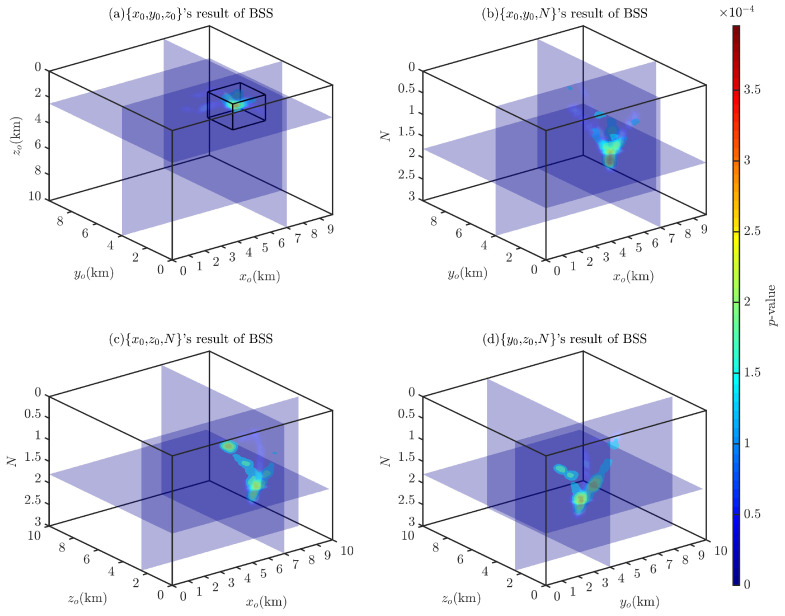
Probability density isosurface obtained using 3D BSSFFT with $\lambda =\left[100,100,100\right]$ for subsets (**a**) $\left\{x_{0},y_{0},z_{0}\right\}$; (**b**) $\left\{x_{0},y_{0},N\right\}$; (**c**) $\left\{x_{0},z_{0},N\right\}$; (**d**) $\left\{y_{0},z_{0},N\right\}$.

Figure 8 and Figure 9 show the Euler solution subsets’ 3D BSS and BSSFFT results, respectively. Through the comparative analysis of these two figures, it can be observed that in the 3D probability density estimation results, with the exception of a subset $\left\{x_{0},y_{0},z_{0}\right\}$, which has a single probability density peak, the results of all subsets show multiple peaks due to spurious solutions, with the most prominent peak corresponding to the theoretical settings of the model. Compared to the BSS result, it can be seen that the BSSFFT method gives more compact results, with only one probability density peak, and is unaffected by the spurious solutions. Since the probability density values corresponding to spurious solutions are very small, as shown in Figure 9, it is convenient to identify the influence of these spurious solutions. By comparing the 1D, 2D, and 3D probability density results, it can be concluded that the probability density peaks/contours/isosurfaces obtained by BSSFFT can be used to analyze Euler solution subsets, avoid the influence of spurious solutions, and effectively determine anomaly sources. Regarding the computation time for the analysis of the 3D subsets $\left\{x_{0},y_{0},z_{0}\right\}$, $\left\{x_{0},y_{0},N\right\}$, $\left\{x_{0},z_{0},N\right\}$, and $\left\{y_{0},z_{0},N\right\}$, the BSS algorithm requires 29.1144, 26.2609, 23.9917, and 32.2006 s, and the BSSFFT algorithm requires 3.4662, 2.6576, 2.3155, and 3.5115 s, respectively. Therefore, BSSFFT is considered to be superior to the BSS algorithm to some extent.

### 4.2. Synthetic Model

**Figure 10:**
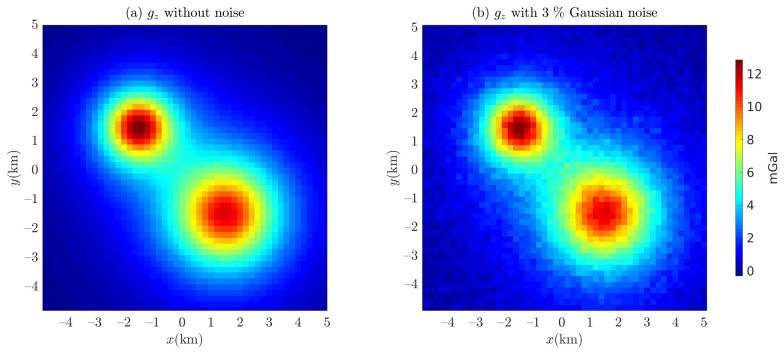
$g_{z}$ of the synthetic model (**a**) without noise and (**b**) with 3% Gaussian noise. The density values of left and right anomalous sources are 0.3 g/cm^3^ and 0.7 g/cm^3^, respectively.

**Figure 11:**
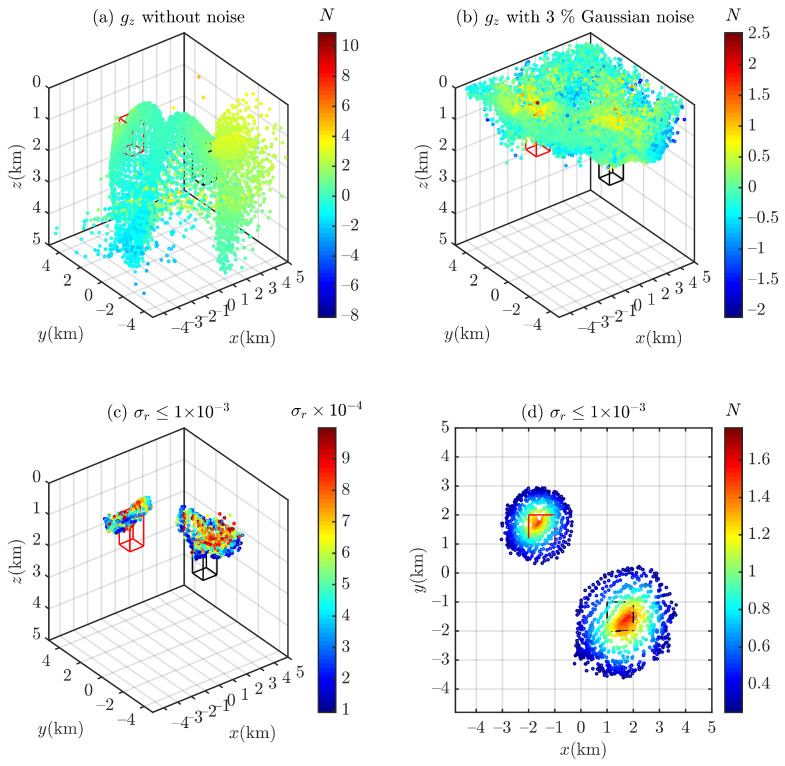
Scatter plots of Euler solutions. The red box and black box are representative of the abnormal source. (**a**) $g_{z}$ without noise and (**b**) with 3% Gaussian noise, Euler solutions filtered by $\sigma_{r}\leq 1\times 10^{-3}$ in different views: (**c**) (−37.5,30) and (**d**) (90,0).

To further verify the correctness of the proposed BSSFFT algorithm for multiple anomalies, based on a single cube model setup, a synthetic model is studied. It is composed of two cubes, both 1000 m × 1000 m × 1000 m in size, with center-of-mass coordinates of (1500, −1500, 2000) and (1500, −1500, 2000). Euler deconvolution with a sliding window of 15×15 is used to process gravity data contaminated by 3% Gaussian noise based on Equation (23) in order to obtain Euler solutions (see Figure 10 and Figure 11).

**Figure 12:**
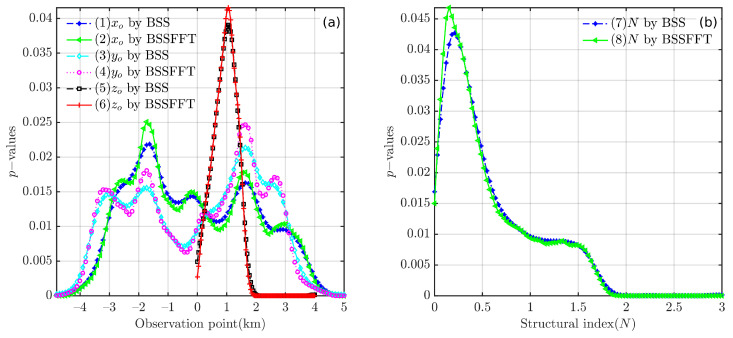
Probability density curves obtained using 1D BSS with λ=100 for subsets of the combination models. (**a**) subsets $\left\{x_{0}\right\}$, $\left\{y_{0}\right\}$, and $\left\{z_{0}\right\}$; (**b**) subset {N}.

As shown in Figure 12, by applying 1D BSS and BSSFFT to the Euler solutions derived from the synthetic model, probability density curves are obtained for each subset of the Euler deconvolution solutions. The 1D BSS and BSSFFT results provide accurate estimates for each subset; this can be seen by comparing the horizontal coordinates of each density peak with the theoretical value of the synthetic model, as shown in Table 1. However, similar to the results in Figure 6, the presence of multiple peaks makes it difficult to effectively use 1D probability density curves to analyze Euler solutions in practical applications.

**Figure 13:**
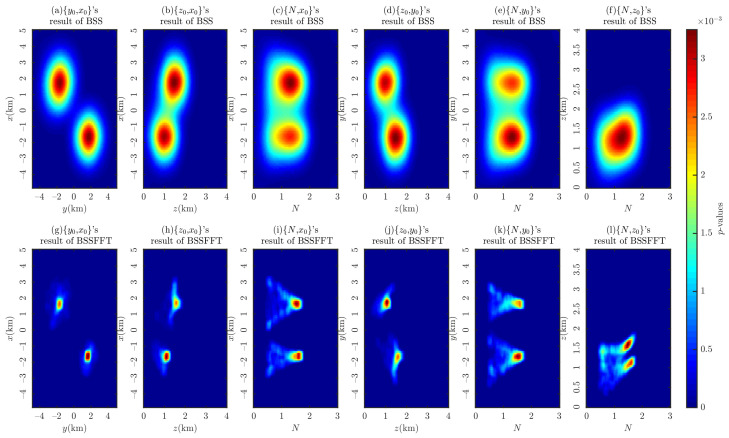
Probability density distribution obtained using 2D BSS and BSSFFT with $\lambda =\left[100,100\right]$ for subsets $\left\{y_{0},x_{0}\right\}$, $\left\{z_{0},x_{0}\right\}$, $\left\{N,x_{0}\right\}$, $\left\{z_{0},y_{0}\right\}$, $\left\{N,y_{0}\right\}$, and $\left\{N,z_{0}\right\}$ of the combination model.

Figure 13 shows the probability density distribution of each subset obtained from the 2D Euler solutions of the combined model using BSS and BSSFFT. By comparing the models’ positions and their structural indices, the 2D BSSFFT results can be used to determine the spatial positions of the anomaly sources and their boundaries, showing good focusing performance compared to the 2D BSS method. However, there is some ambiguity due to the model setup, i.e., both the cubic burial depths and structural indices are the same, resulting in only one peak in Figure 13f, as shown in Table 2. For this reason, 3D Euler solution subsets analyzed with the synthetic model using only the 3D BSSFFT are considered.

**Figure 14:**
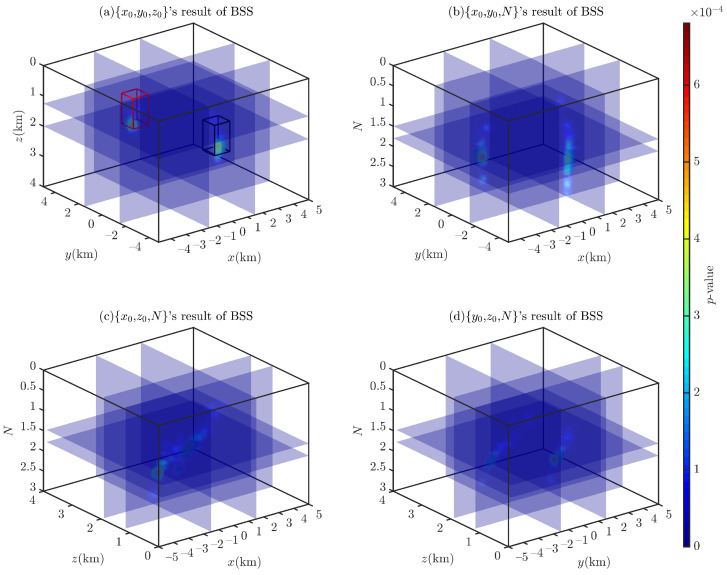
Probability density isosurface obtained using 3D BSSFFT with $\lambda =\left[100,100,100\right]$ for subsets (**a**) $\left\{x_{0},y_{0},z_{0}\right\}$, (**b**) $\left\{x_{0},y_{0},N\right\}$, (**c**) $\left\{x_{0},z_{0},N\right\}$, and (**d**) $\left\{y_{0},z_{0},N\right\}$ of the combination model. The red box and black box are representative of the abnormal source.

Figure 14 shows the 3D BSSFFT density estimation results for the subset of the synthetic model. Comparing the estimation results of the 1D, 2D, and 3D BSS, and BSSFFT, it can be observed that the BSSFFT method produces more compact results, with only one density peak unaffected by the instabilities of the Euler homogeneity equation. The results are remarkably stable, with very few instances of probability density tails [7] that correspond to spurious solutions.

From Table 1, Table 2 and Table 3, it can be seen that the results of the probability density estimation for the two cubes are slightly shifted from the theoretical values. This phenomenon is visualized in Figure 14, particularly in Figure 14a. Through a comparison with Figure 9, it can be hypothesized that the mutual interference of adjacent anomalous sources causes this.

A comprehensive analysis of the above results indicates that the BSSFFT can effectively localize anomaly sources. Therefore, in our subsequent research, only the BSSFFT method proposed in this paper will be used to analyze Euler solutions.

### 4.3. Sensitivity of 3D BSSFFT to Separations

To evaluate the performance of the proposed algorithms in distinguishing neighboring anomaly sources, three models presented are used in the work by Cao et al. [37], each consisting of two cubes of the same size (side length of 2.0 (km)) and different densities (2.36 g/cm^3^ and 1.27 g/cm^3^ for the left and right cubes, respectively), at separation L = 4.0 (km), 2.5 (km), and 1.0 (km), respectively. These values of separation L allow the two sources to approach each other simultaneously in the *x* and *y* directions. Table 4 lists the two cubes’ parameters. A subset of $\left\{x_{0},y_{0},z_{0}\right\}$, obtained from FTG data and contaminated by 3% Gaussian noise based on Equation (23), is used in this 3D BSSFFT study.

**Figure 15:**
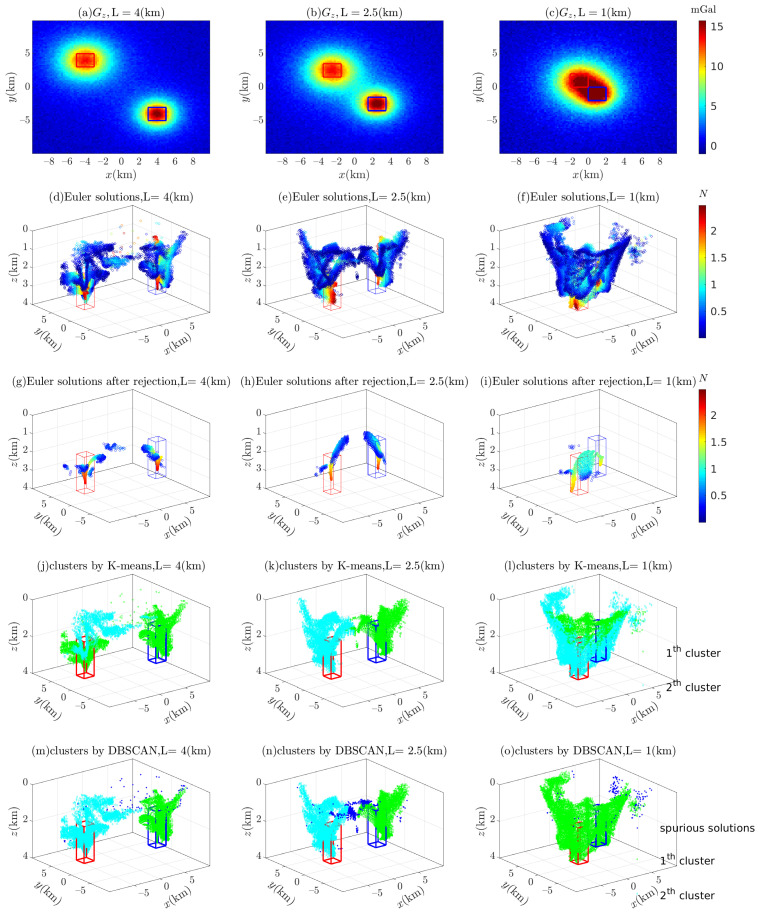
Clustering methods used to separate the adjacent clusters formed by Euler solutions. The red box and blue box are representative of the abnormal source. (**a**–**c**) Contaminated gravity; (**d**–**f**) Euler solutions obtained using $w_{x}\equiv w_{y}=3∼15$; (**g**–**i**) Euler solutions after rejection by $\sigma_{r}\leq 1\times 10^{-3}$; (**j**–**l**) clusters obtained via the K-means clustering algorithm (K-means), where the number of clusters is predetermined by 2; and (**m**–**o**) three clusters automatically obtained using the density-based spatial clustering of applications with noise (DBSCAN) algorithm, setting the number of targets in their neighborhood to 1% of the total number of samples. There are 32,362, 42,237, and 48,266 solutions after filtering in (**g**–**i**), respectively.

As the separation L decreases, it becomes more difficult to distinguish between two adjacent cubes using the Euler solutions in Figure 15d–f, even with Thompson’s empirical formula [4], as shown in Figure 15g–i. Moreover, as seen in Figure 15j–l, similar to the fuzzy C-means clustering algorithm (FCM) [37], the k-means clustering algorithm (K-means) depends on the number of predefined clusters; thus, it can effectively discriminate between adjacent anomaly sources but cannot eliminate spurious solutions. On the other hand, some spurious solutions, such as the green Euler solutions in Figure 15m–o, can be found by using the density-based spatial clustering of applications with noise (DBSCAN) algorithm, when the distance between nearby anomaly sources is relatively large. Regarding the computation time for different values of L (4.0 (km), 2.5 (km), and 1.0 (km)), the K-means algorithm requires 0.1223, 0.1927, and 0.2839 s, and the DBSCAN algorithm requires 69.2537, 64.1225, and 72.1126 s, respectively.

**Figure 16:**
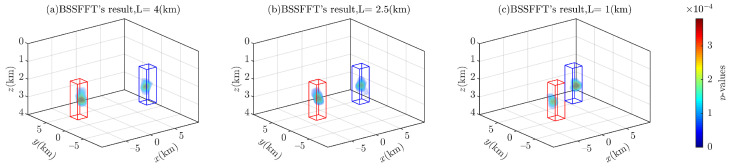
Probability density isosurfaces obtained using 3D BSSFFT for subset $x_{0},y_{0},z_{0}$ with varied separations L: (**a**) 4.0 (km); (**b**) 2.5 (km); and (**c**) 1.0 (km); $\lambda =\left[100,100,100\right]$. The red box and blue box are representative of the abnormal source.

Figure 16 illustrates the separation of the anomaly sources by using 3D BSSFFT to estimate the subset $\left\{x_{0},y_{0},z_{0}\right\}$. Unlike other techniques used to separate adjacent anomaly sources, the two probability density peaks directly distinguish two sources at different separations L, as shown in Figure 15.

### 4.4. Sensitivity of 3D BSSFFT to Gaussian Noise

If the signal-to-noise ratio of the input data is low, the Euler solutions will tend to diverge. Many strategies/methods have been proposed to eliminate spurious solutions and determine the optimal solutions [4,7,43]. For example, clustering methods cannot effectively distinguish spurious solutions from shallow sources and neighboring bodies [15,59]. Therefore, a model containing two cubes with a separation L of 2.5 (km) (see Figure 16) is selected, and the corresponding Euler solutions are derived from the corresponding forward data, which are contaminated with $\bar{p}=$ 0%, 4%, and 8% noise based on Equation (23). On this basis, the sensitivity of the BSSFFT method to different levels of Gaussian noise is analyzed.

**Figure 17:**
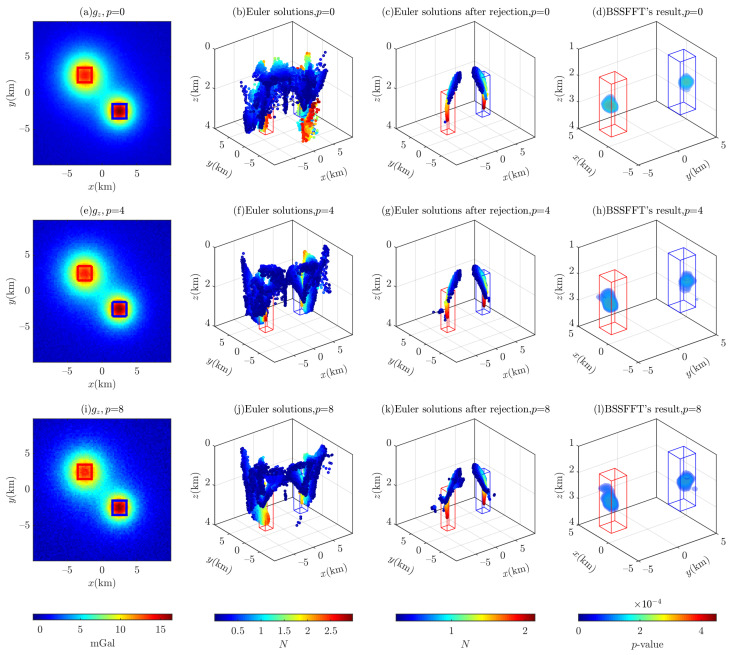
Illustration of the 3D BSSFFT method’s sensitivity to different levels of Gaussian noise. The red box and blue box are representative of the abnormal source. The top, middle, and bottom rows correspond to $\bar{p}=$ 0%, 4%, and 8%, respectively; the columns from left to right correspond to noise-corrupted gravity, Euler solutions, Euler solutions filtered by $\sigma_{r}\leq 1\times 10^{-3}$, and the probability density distributions obtained from the BSSFFT with $\lambda =\left[100,100,100\right]$, respectively. $w_{x}\equiv w_{y}=3∼15$. There are 43,258, 42,552, and 48,923 solutions after filtering in (**c**,**g**,**k**), respectively.

As shown in Figure 17, the contours in the gravity map become increasingly distorted as the percentage of noise increases, and the number of Euler solutions with low SIs increases dramatically. However, this is not obvious in Figure 17b,e,h or Figure 17c,g,k, due to the fact that the previously drawn solutions overlap with the later ones, and backward solutions are obscured by forward ones. Compared to the results in Cao et al. [37], as we use a series of sliding windows of different sizes to generate the Euler solutions, the estimated positions derived from the 3D probability density distribution are very close to the theoretical positions of the synthetic model, despite the percentage of noise being equal to 8%. However, the estimated positions are all far from the center of the anomaly sources due to the influence of neighboring anomaly sources.

### 4.5. Sensitivity of 2D Euler Deconvolution to Gaussian Noise

**Figure 18:**
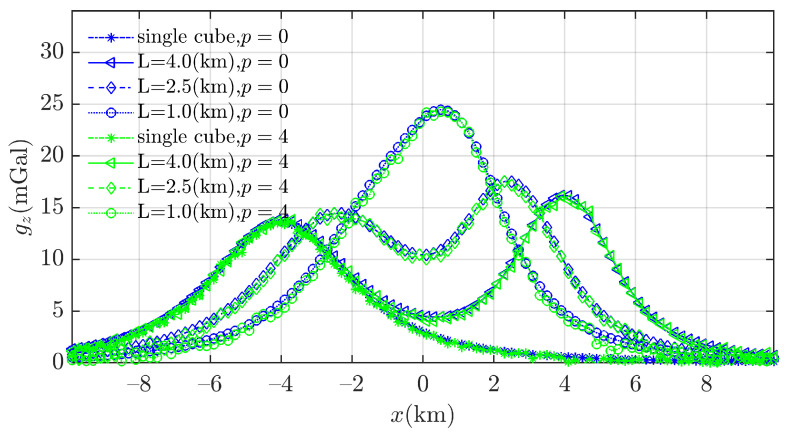
The profiles $g_{z}$ of single and two anomalous sources at different noise levels (p=0,4), respectively.

**Figure 19:**
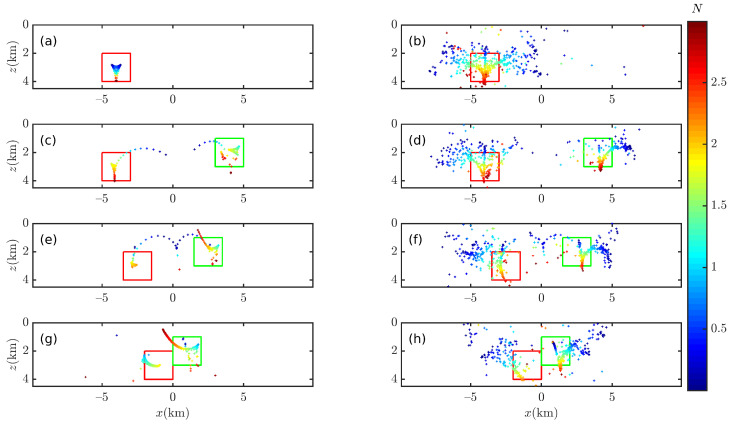
Illustration of the 2D Euler deconvolution sensitivity to different levels of Gaussian noise. The red box and green box are representative of the abnormal source. The left (**a**,**c**,**e**,**g**) and right (**b**,**d**,**f**,**h**) columns correspond to $\bar{p}=$ 0% and 4%, respectively; each row from top to bottom corresponds to the single cube and two cubes with L = 4.0 (km), 2.5 (km), and 1.0 (km), respectively. The density of left and right anomalous sources is 2.36 g/cm^3^ and 1.27 g/cm^3^, respectively. $w_{x}=10∼35$.

The two cubes described in Table 4 were placed on the *y*-axis to construct four models to verify the correctness of the 2D Euler deconvolution algorithm, as shown in Figure 18 and Figure 19.

The Euler solutions are obtained by means of Euler deconvolution in conjunction with the gravity analytic solution of the rectangular, as shown in Figure 19. When the data are free of noise, in Figure 19a,d,g,j, the Euler deconvolution yields “pure” Euler solutions for a single cube model; for two cube models, as L decreases, it is clearly seen that most of the Euler solutions are in the tails and have deviated from the anomaly source. When the data are contaminated with noise (p=4%), the 2D Euler deconvolution can no longer accurately obtain the optimal solutions, and only part of the Euler solution is inside the anomalous source; however, when the two anomalous sources are close to each other (L = 1.0 (km)), the 2D Euler deconvolution has detected a deeper anomalous source, but the Euler solution is farther away from its center.

**Figure 20:**
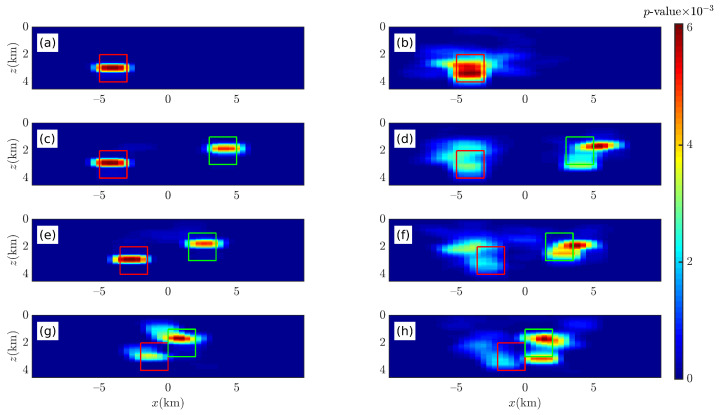
Probability density distribution obtained using 2D BSSFFT with $\lambda =\left[100,100\right]$ for subsets $\left\{x_{0},z_{0}\right\}$ of the single cube and two cubes with L = 4.0 (km), 2.5 (km), and 1.0 (km), respectively. The red box and green box are representative of the abnormal source. The left (**a**,**c**,**e**,**g**) and right (**b**,**d**,**f**,**h**) columns correspond to $\bar{p}=$ 0% and 4%, respectively.

Compared with the results of 2D Euler deconvolution (see Figure 19), the results of 2D BSSFFT can locate the anomaly source more clearly; compared with 3D BSSFFT’s results (see Figure 16 and Figure 17d,h,l), the estimation results of 2D BSSFFT may deviate from the center of the anomaly source when the noise level is high or when adjacent anomaly sources are close to each other, as shown in Figure 20.

## 5. Bishop Model

To improve the algorithm’s credibility, we introduce a widely used model, i.e., the Bishop model. It is a 3D synthetic basement model [60], which was first proposed by Fairhead et al. [61]. The terrain data used are derived from a part of the digital elevation model in the volcanic highlands of northern Bishop, California, covering an area of about 10.5 km ×10.5 km, scaled and shifted in the depth direction, i.e., has been upscaled by a factor of 30 in the *x*, *y*, and *z* dimensions and then subtracted from a fixed value, resulting in the surface layer being translated below sea level [62,63]. Then, a 3D basement model with a horizontal grid size of 500 m × 500 m is created using the terrain data [63]. Because there are two large offset faults in this area [64], the elongated deep tectonic low pressure and other minor faults are formed from west to east and from north to south, and the top structure of the magnetic base layer similar to the basement is further formed [64,65].

Based on Bishop’s 3D basement model, Fairhead et al. [61] used the Northwest Geophysical GM-SYS 3D software (version 4.10) to calculate and obtain the gravity and magnetic field forward results, thus constructing the Bishop 5X dataset [66,67]. Because it is a shared complex gravity/magnetic anomaly dataset, it is welcomed and studied by many researchers in the field of geophysics [60,61,65,66,67,68,69,70].

The Bishop 5X dataset contains only gravity anomalies derived from strata and complex magnetic anomalies. Therefore, to obtain the corresponding pseudo-gravity anomaly, Ekinci and Yigitbas [71] used pseudo-gravity transformation to convert magnetic data into pseudo-gravity data with gravity data characteristics to assist geological interpretation [71,72,73]. Pseudo-gravity transformation [72] used Fourier technology [74] as a “bridge” to integrate the grid data of total magnetic field intensity to obtain pseudo-gravity grid data [71,75]. The pseudo-gravity data can reduce the advantages of the shallow magnetic source and enhance the magnetic anomaly related to the deep magnetic source. Because pseudo-gravity data represent intuitive depictions of the underground magnetic field, they are not directly related to gravity anomalies or density. Essentially, they pertain to magnetic anomalies, which can only be explained by the distribution of magnetization [76,77]. Therefore, the data are rarely valued and applied in practical exploration problems [72,78]. Thus, we apply the Euler deconvolution Equations (1) and (2) to the magnetic data of the Bishop model. Considering that the data point interval of the Bishop 5X dataset is much smaller than the magnetic sources’ size, we use 1000 m × 1000 m to re-grid the Bishop 5X magnetic grid data.

**Figure 21:**
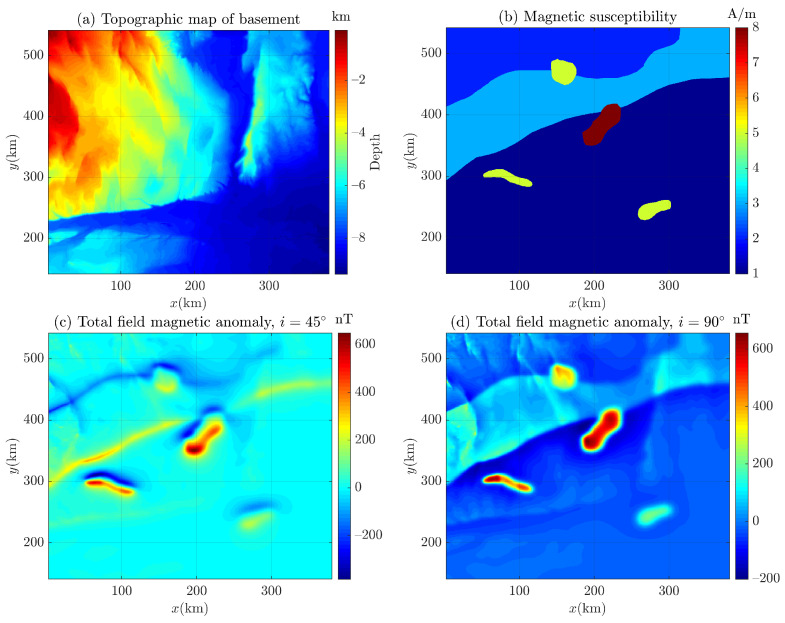
The Bishop 5X dataset, (**a**) topographic map of Basement, (**b**) magnetic susceptibility, (**c**) total field magnetic anomaly, $i=45^{\circ }$, and (**d**) total field magnetic anomaly, $i=90^{\circ }$.

**Figure 22:**
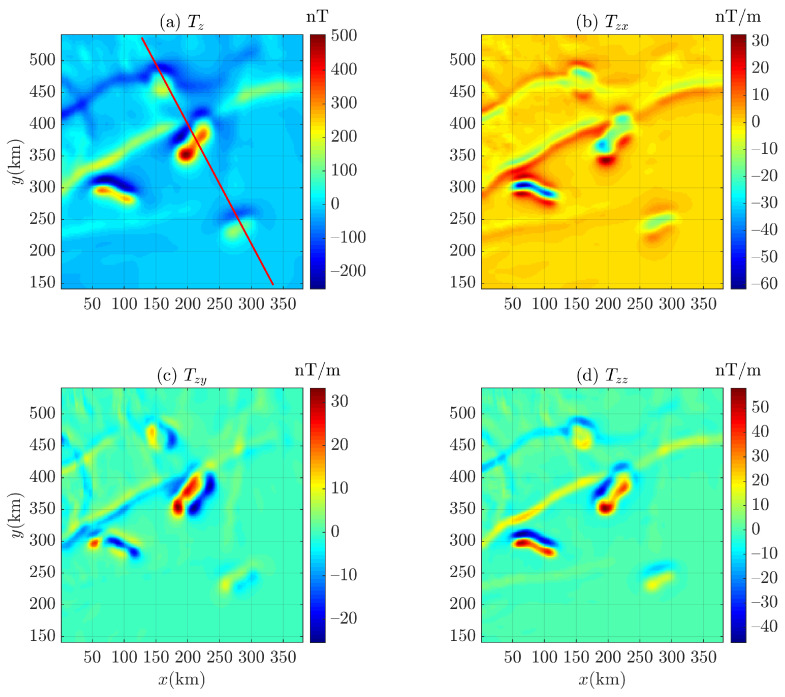
The components (**a**) $T_{z}$ and first-order derivatives (**b**) $T_{zx}$, (**c**) $T_{zy}$, and (**d**) $T_{zz}$ of the magnetic anomaly (magnetic inclination $45^{\circ }$) of Bishop 5X data. The red line is the survey line applying the 2D Euler deconvolution.

**Figure 23:**
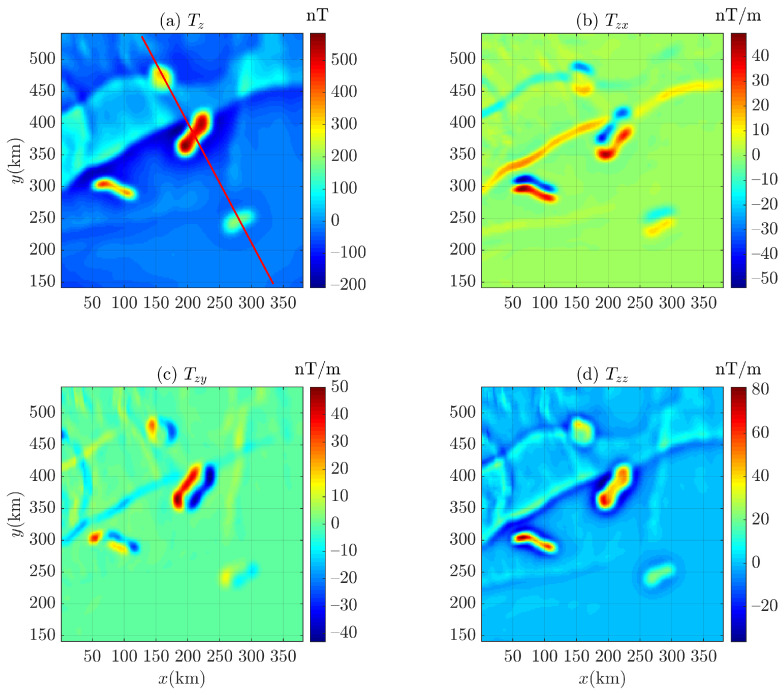
The components (**a**) $T_{z}$ and first-order derivatives (**b**) $T_{zx}$, (**c**) $T_{zy}$, and (**d**) $T_{zz}$ of the magnetic anomaly (magnetic inclination $90^{\circ }$) of Bishop 5X data. The red line is the survey line applying the 2D Euler deconvolution.

Considering that it is difficult to obtain the background field information of an input field data by Euler deconvolution, we use correlation analysis to determine the background field [37,79,80,81] and obtain the corresponding residual magnetic anomaly field (i.e, the residual field between the magnetic anomaly and the background field, as shown in Figure 21c,d) for the magnetic anomaly of Bishop 5X (the magnetic inclination $i=45^{\circ }$ and $i=90^{\circ }$), as shown in Figure 21. To distinguish between gravity data and magnetic data in this paper, the marker *g* of Equations (1) and (2) is changed to *T* for magnetic data. Then, the residual magnetic anomaly (also denoted as $T_{z}$ in this paper) is converted into corresponding first-order derivatives ($T_{zx}$, $T_{zy}$, and $T_{zz}$), as shown in Figure 22 and Figure 23. It can be seen that there is no data distortion or oscillation in each subgraph.

**Figure 24:**
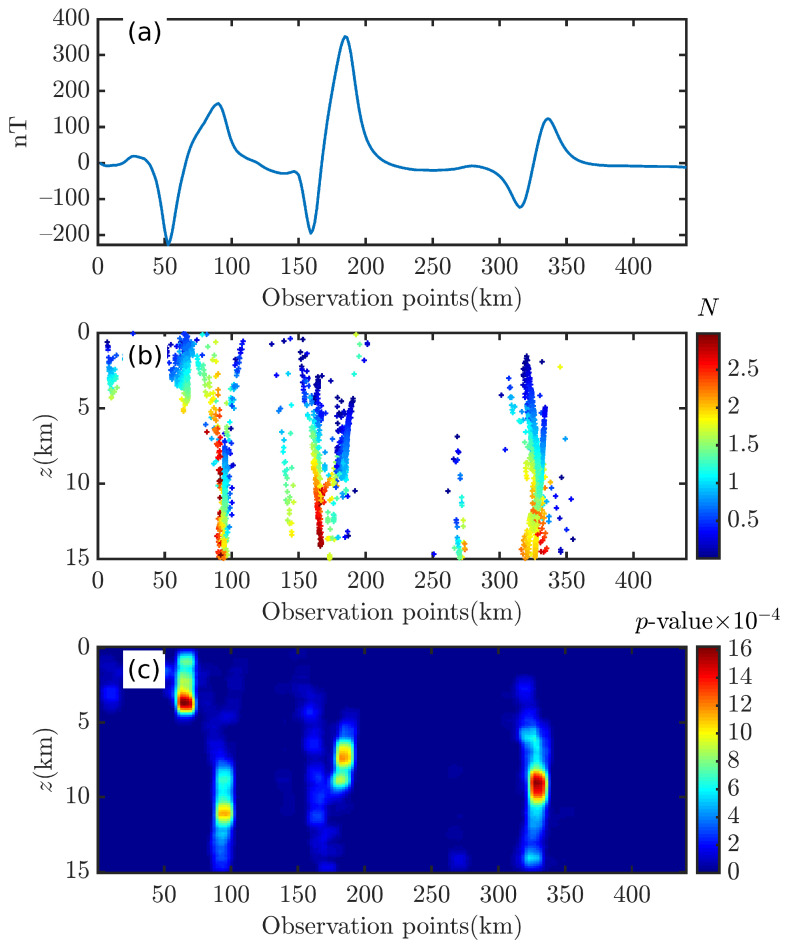
Illustration of the 2D BSSFFT method for Bishop 5X profile data ($i=45^{\circ }$). (**a**) $T_{z}$, (**b**) Euler solutions, (**c**) BSSFFT’s result. The computation time of the 2D Euler deconvolution and BSSFFT is 12.19 and 0.0253 s, respectively. $n=7220,\lambda =\left[100,100\right]$.

**Figure 25:**
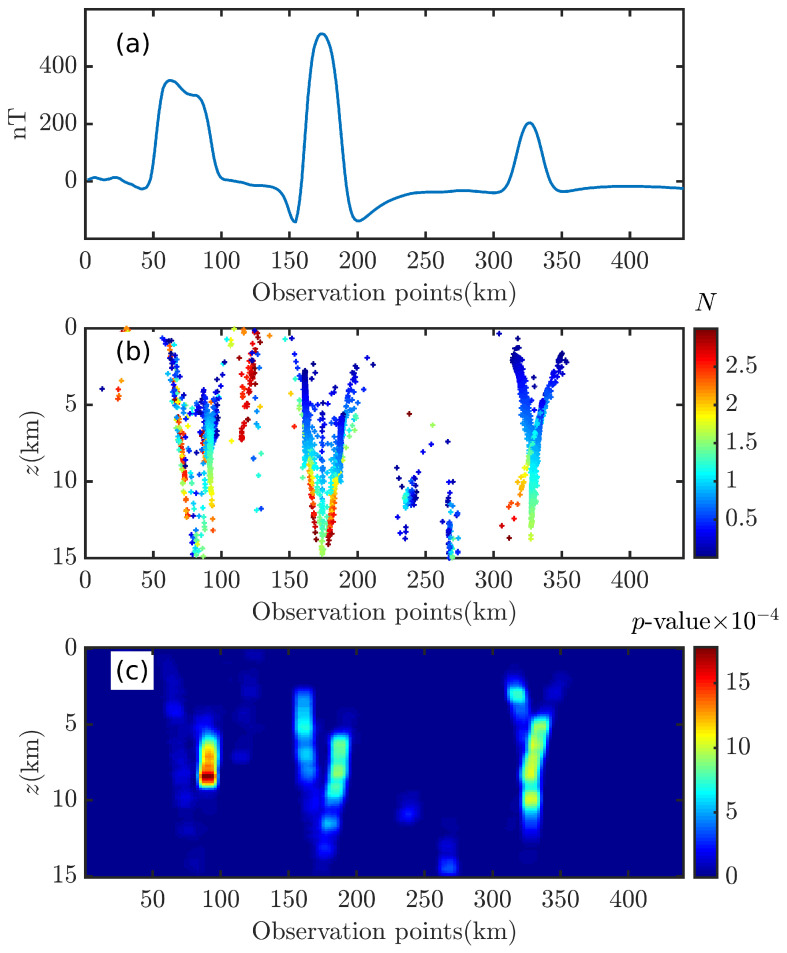
Illustration of the 2D BSSFFT method for Bishop 5X profile data ($i=90^{\circ }$). (**a**) $T_{z}$, (**b**) Euler solutions, (**c**) BSSFFT’s result. The computation times of the 2D Euler deconvolution and BSSFFT are 11.25 and 0.0372 s, respectively. $n=7019,\lambda =\left[100,100\right]$.

Here, it is assumed that the magnetic sources’ spatial distribution information is unknown. For this reason, we use Equations (1) and (2) in 2D and 3D space with different-sized sliding windows ($w_{x}=10∼35$) to obtain the corresponding Euler solutions. For the 2D application, a red survey line spanning three sources of anomalies is selected, and $T_{z}$ and its corresponding derivatives are obtained by Fourier transform [82], as shown in Figure 22a and Figure 23a. By comparing and analyzing Figure 24 and Figure 25, it can be seen that both Euler solutions show a relatively scattered trend, and only the optimal solutions may be seen to show a linear aggregation trend. However, under the interference of spurious solutions, it is difficult to determine optimal solutions and use them to locate the source of anomalies. In contrast, the BSSFFT’s results are effective in identifying the anomaly source with comparable depth resolution. Compared to Figure 25c, there is a probability density peak interference in the shallow part of Figure 24c, which we hypothesize is related to the interference caused by the magnetic inclination.

**Figure 26:**
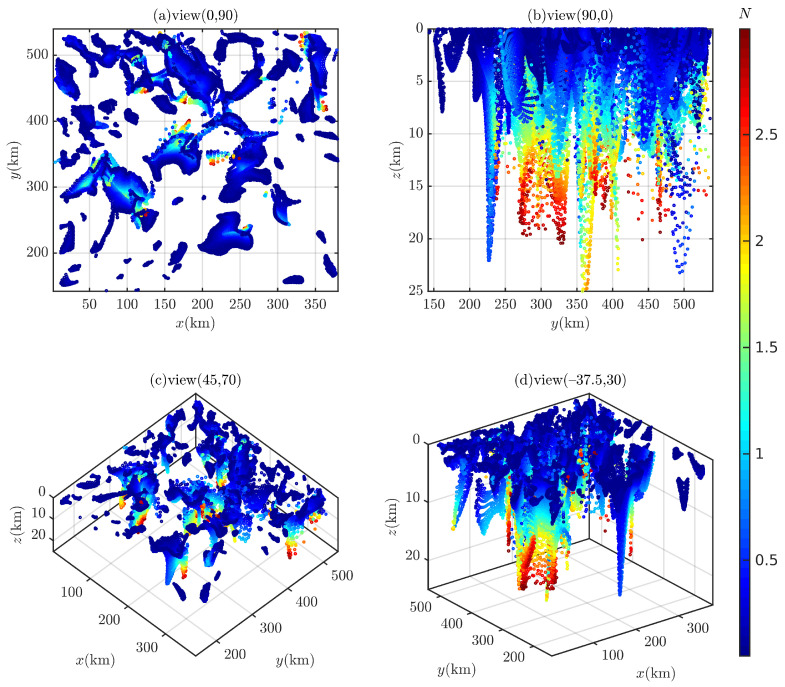
The Euler solutions of Bishop 5X (magnetic inclination $45^{\circ }$).

**Figure 27:**
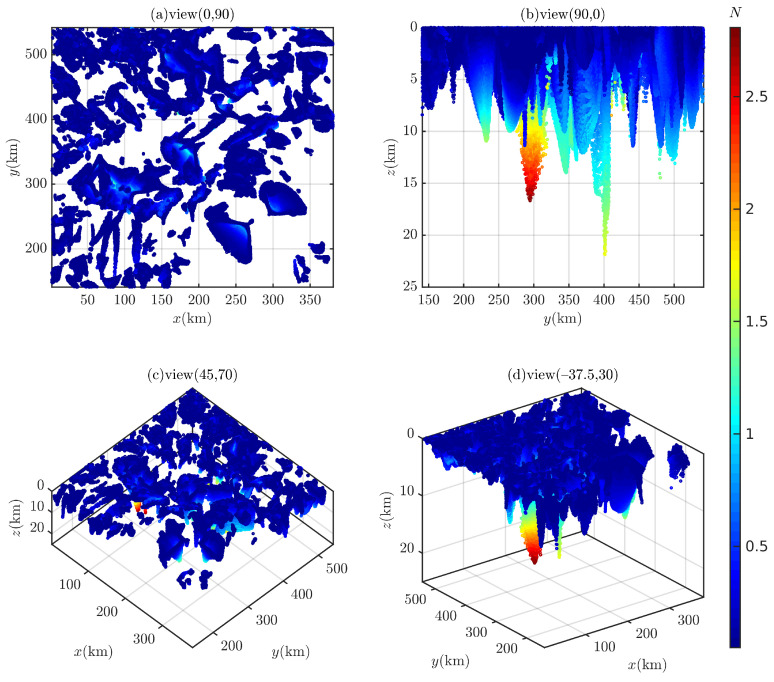
The Euler solutions of Bishop 5X (magnetic inclination $90^{\circ }$).

In the 3D case, for $i=45^{\circ }$ and $90^{\circ }$, the computation time required for the Euler deconvolution to traverse the entire survey area is 572.25 s and 601.39 s, respectively. A total of 237,292 and 286,370 Euler solutions are obtained (filtered by N<0.25 or N>3.0), as shown in Figure 26 and Figure 27. A large number of Euler solutions with small *N* values are scattered in the shallow part, which are likely to be spurious solutions. The structural index *N* increases continuously as depth *z* increases, and when z>15,000 m, Euler solutions (N>2.5) are somewhat distorted. In Figure 26b,d and Figure 27b,d, with the help of geological boundary lines, some Euler solutions can mark magnetic sources. However, these Euler solutions cannot be distinguished from other scattered Euler solutions.

For this reason, we use the BSSFFT algorithm with an estimated grid of 100×100×50 to perform probability density analysis on these Euler solutions in Figure 26 and Figure 27. For $i=45^{\circ }$ and $90^{\circ }$, the calculation times are 3.0502 and 3.1829 s, respectively.

**Figure 28:**
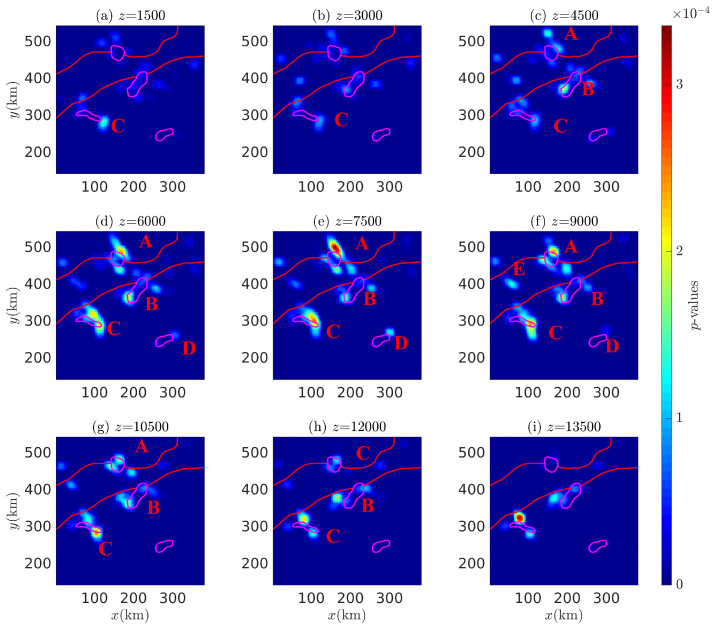
Probability density slices of the Euler solution at various depths: (**a**) 1500, (**b**) 3000, (**c**) 4500, (**d**) 6000, (**e**) 7500, (**f**) 9000, (**g**) 10,500, (**h**) 12,000, and (**i**) 13,500 (magnetic inclination $i=45^{\circ }$). A–D are denoted as probability density peaks.

**Figure 29:**
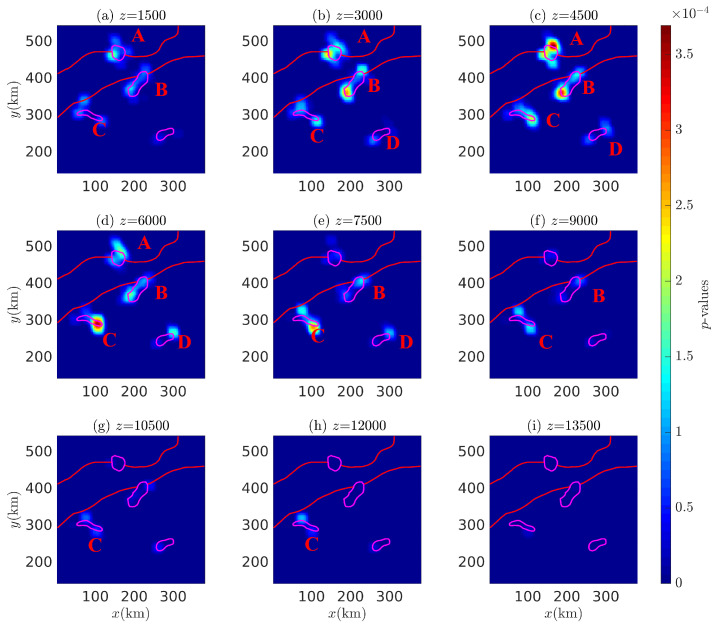
Probability density slices of the Euler solution corresponding to (**a**) 1500, (**b**) 3000, (**c**) 4500, (**d**) 6000, (**e**) 7500, (**f**) 9000, (**g**) 10,500, (**h**) 12,000, and (**i**) 13,500 (magnetic inclination $i=90^{\circ }$). A–D are denoted as probability density peaks.

The different depth probability density slices of the Euler solutions for the Bishop 5X magnetic anomaly with $i=45^{\circ }$ and $90^{\circ }$ are shown in Figure 28 and Figure 29, showing multiple density peaks in these subfigures. Peak *A* consists of several sub-peaks surrounding adjacent anomalous sources, which, combined with stratigraphic information, suggests that peak *A* may be associated with adjacent faults and strata; peak *B* extends from z=4500 m down to 13,500 m, but fails to accurately indicate the corresponding magnetic source; peak *D* extends from z=4500 m down to z=7500 m, but occurs only at the southwestern end of the corresponding anomalous source and affects a limited depth range. In contrast to the other peaks associated with the corresponding anomalous sources, peak *C* extends from z=1500 m down to z=7500 m, pinpointing the source at z=4500∼6000 m (at the maximum probability density value).

The correlation between the probability density peaks in Figure 29, the geological structure boundary, and the magnetic anomaly sources is similar to that in Figure 28. However, peaks *B* and *E* are different. In Figure 29, peak B extends downward from z=3000 m to z=7500 m, showing two separate peaks, similar to the two peaks of the cylinder model affected by the equivalent effect described in reference Cao et al. [37]. Compared to Figure 29, Figure 28 shows a large number of very small amplitude probability dense peaks that fail to dominate any subgraph. It can be concluded that these peaks are due to the boundary effect of the survey area and the influence of magnetic inclination.

**Figure 30:**
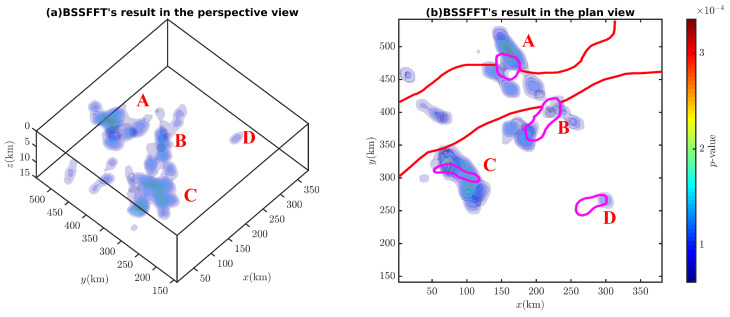
Probability density isosurfaces derived from Bishop 5X magnetic data ($i=45^{\circ }$) in (**a**) the perspective view and (**b**) the plane view (from bottom to top). A–D are denoted as probability density peaks.

**Figure 31:**
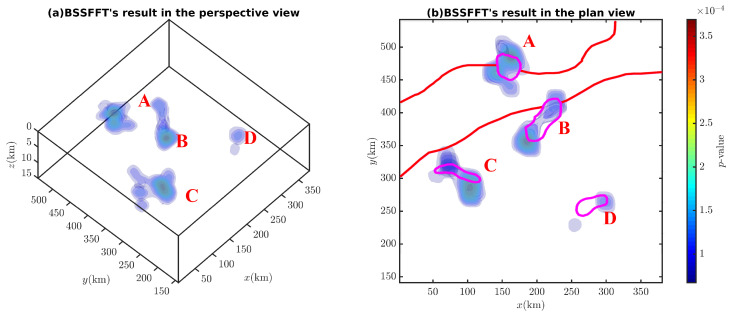
Probability density isosurfaces derived from Bishop 5X magnetic data ($i=90^{\circ }$) in (**a**) the perspective view and (**b**) the plane view (from bottom to top). A–D are denoted as probability density peaks.

Figure 30 and Figure 31 show that the corresponding probability density isosurfaces of the magnetic anomaly data obtained by the proposed method can effectively determine the anomaly source’s spatial distributions while avoiding the influence of magnetic inclination (see Figure 29). Referring to Cao et al. [37], more focused results will be obtained if smaller bandwidths or larger estimation grids are used.

## 6. Discussion

In practice, violations of the Euler homogeneity conditions (e.g., inappropriate data grid spacing), low signal-to-noise ratios (either natural or FFT-induced) [7,9,42,83,84], and source overlay have resulted in many spurious solutions, leading to a sparsely dispersed distribution of the entire Euler solution set, as opposed to a dense distribution [9,83,84,85]. When interpreting Euler solutions, the abundance of solutions generated by Euler deconvolution leads to rendering issues where previously drawn solutions overlap with later ones, and backward solutions are obscured by forward ones. Therefore, the end goal of the processing and interpretation of Euler solutions is not merely to reject spurious solutions [39,42,44,84]. To overcome the above problems, we propose the BSS and BSSFFT methods based on probability density estimates, which are used to apply Euler solutions for more intuitive geological interpretation.

The apparent *N* may appear to be different due to numerical issues, but the true value is always constant [37,40]. Therefore, in practical applications, Euler deconvolution uses a series of predefined structural indices, which leads to the presence of many solutions in the Euler deconvolution process [6,10]. Since it is challenging to determine *N* for the geological target of interest [41,42], this work adopts the Euler deconvolution of gravity/magnetic data with an unprescribed structural index to obtain the Euler solutions. Furthermore, as fixed-size sliding windows cannot effectively identify anomaly sources of varying sizes, the Euler deconvolution of gravity data in conjunction with differently sized sliding windows is applied to the field data.

For a given sample, the bandwidth is inversely proportional to the grid size. The bandwidth determines the level of smoothness in the resulting probability density curve, reflecting the proportion of observed data points contributing to the formation of the curve. A larger bandwidth results in a flatter curve due to a lesser contribution of individual data points to the final shape; conversely, a smaller bandwidth leads to a steeper curve because of a greater contribution of the data points. Therefore, the quality of the BSS’s final probability density distribution is influenced by the bandwidth parameter or the grid size. If the sample size is not considered, the memory consumption of the BSS and BSSFFT algorithms depends solely on the size of the estimation grid and the peak memory usage of the n-dimensional FFT. Therefore, the memory consumption of BSSFFT is higher than that of BSS.

Increasing the grid size significantly reduces the computational efficiency of BSS, particularly for large data samples. Furthermore, combining sample data with high-dimensional sparse data, especially in scenarios involving multiple-density clusters, complicates the efficient identification of the “best” bandwidth [86].

In this study, to effectively overcome these challenges associated with increased computational efficiency, BSSFFT is implemented using fast linear binning approximation and FFT technology, which significantly improve the computational efficiency in the estimation of BSS [37]. Moreover, experimenting with grids of different sizes or selecting isosurfaces from different formations could be more effective in identifying interesting geological structures.

For adjacent anomaly sources, the results obtained using FCM, DBSCAN, and probability density were compared and analyzed, and 3D probability density contours/isosurfaces could effectively separate adjacent anomaly sources [37]. Due to the fast linear binning approximation and FFT techniques, we can apply different grid sizes and/or select different formations of isosurfaces to identify interesting geological structures more efficiently. For 3D applications, the 1D or 2D subsets produce too many probability density distribution curves or images, making simultaneous interpretation difficult. The 5D results $\left\{x_{0},y_{0},z_{0},N,p-value\right\}$ are obtained using BSSFFT for Euler solutions but are challenging to demonstrate.

In this work, we use BSSFFT to analyze the subsets $\left\{x_{0},z_{0}\right\}$ (2D case) and $\left\{x_{0},y_{0},z_{0}\right\}$ (3D case) obtained by the conventional Euler deconvolution with some success. By analyzing the 4D probability density distribution, we can obtain information with a good depth and lateral resolution for the analysis of the geological structures in the study area.

## 7. Conclusions

This study addresses the optimization of the computational efficiency problem found in a previous work. Inspired by the work of Wand [32], fast linear binning approximation with FFT technology is applied, which significantly improves the efficiency in probability density estimation. The current study uses fast linear binning approximation and FFT technology to provide a quick and efficient BSSFFT algorithm. Then, by analyzing the computational accuracy of KS, BSS, and BSSFFT, it is concluded that BSSFFT has high computational accuracy. Supported by the fast linear binning approximation technique, BSSFFT demonstrates strong focusing capabilities.

The procedure of the BSSFFT method for representing geological bodies using Euler solutions, as proposed in this paper, bears similarities to the KDDE algorithm process. It involves selecting moving windows of varying or fixed sizes and using Equation (2) to traverse grid data for Euler solutions. An estimation grid is constructed with a predefined size λ or bandwidth *h*. The subset $\left\{x_{0},y_{0},z_{0}\right\}$ is projected onto this grid using a fast linear binning approximation to produce a grid count *C*. Using Equation (15) to perform the kernel function evaluation, the process constructs zero-padded versions of the grid count and the kernel evaluation. Fast convolution between these elements via the FFT yields the probability density distribution of the subset $\left\{x_{0},y_{0},z_{0}\right\}$.

The tests with Bishop 5X data and synthetic models show that the BSSFFT approach presented in this paper, in conjunction with subset $\left\{x_{0},y_{0},z_{0}\right\}$ derived from the Euler deconvolution, achieves meaningful geological results that are independent of pre-existing geological data.

## Figures and Tables

**Table 1:** The 1D probability density estimation results.

	Cube 1 (−1500,−1500,2000)				Cube 2 (1500,1500,2000)			
**1D Subset**	$x_{0}$	$y_{0}$	$z_{0}$	N	$x_{0}$	$y_{0}$	$z_{0}$	N
Theoretical values	−1500	−1500	2000	2	1500	1500	2000	2
BSS results	−1632	−1731	1051	1.33	1634	1631	1051	1.33
BSSFFT’s results	−1731	−1731	1051	1.40	1634	1668	1052	1.40

**Table 2:** The 2D probability density estimation results.

2D Subset	$\left(x_{0},y_{0}\right)$	$\left(z_{0},x_{0}\right)$	$\left(N,x_{0}\right)$	$\left(z_{0},y_{0}\right)$	$\left(N,y_{0}\right)$	$\left(N,z_{0}\right)$
BSS results for Cube 1	(1724,−1591)	(1494,1784)	(1.33,1754)	(1515,−1644)	(1.32,−1664)	(1.3,1275) *
BSS results for Cube 2	(−1584,1892)	(1041,−1644)	(1.34,−1674)	(1041,1724)	(1.35,1655)	(1.3,1275) *
BSSFFT results for Cube 1	(1605,−1642)	(1598,1605)	(1.58,1575)	(1639,−1703)	(1.58,−1644)	(1.50,1580)
BSSFFT results for Cube 2	(−1614,1690)	(1103,−1733)	(1.62,−1644)	(1103,1665)	(1.60,1605)	(1.58,1129)

* Unable to separate adjacent anomalies.

**Table 3:** The 3D BSSFFT results.

	$\left\{x_{0},y_{0},z_{0}\right\}$	$\left\{x_{0},y_{0},N\right\}$	$\left\{x_{0},z_{0},N\right\}$	$\left\{y_{0},z_{0},N\right\}$
Cube 1	(1472,−1901,2200)	(1386,−1860,1.98)	(1371,2200,1.89)	(−1874,2280,2.01)
Cube 2	(−1860,1404,1600)	(−1860,1324,2.04)	(−1963,1640,2.01)	(1329,1680,2.10)

**Table 4:** The geometric parameters of the three synthetic models with various separations.

Separation L	Centroid of Left Cube	Centroid of Right Cube
4000	(−4000,4000,3000)	(4000,−4000,2000)
2500	(−2500,2500,3000)	(2500,−2500,2000)
1000	(−1000,1000,3000)	(1000,−1000,2000)

## Data Availability

Data are contained within the article.

## Abbreviations

The following abbreviations are used in this manuscript:

KDDE	kernel density derivative estimation
BSS	B-spline probability density estimation method
BSSFFT	B-spline probability density estimation method based on fast Fourier transform
KS	Gaussian kernel smoothing estimation
FFT	fast Fourier transform
pdf	probability density function
DBSCAN	density-based spatial clustering of applications with noise
K-means	k-means clustering algorithm
FCM	fuzzy C-means clustering algorithm
